# Nucleotide patterns aiding in prediction of eukaryotic promoters

**DOI:** 10.1371/journal.pone.0187243

**Published:** 2017-11-15

**Authors:** Martin Triska, Victor Solovyev, Ancha Baranova, Alexander Kel, Tatiana V. Tatarinova

**Affiliations:** 1 Children’s Hospital Los Angeles, University of Southern California, Los Angeles, CA, United States of America; 2 Faculty of Advanced Technology, University of South Wales, Pontypridd, Wales, United Kingdom; 3 Softberry, Inc. Mount Kisco, NY, United States of America; 4 School of Systems Biology, George Mason University, Fairfax, VA, United States of America; 5 Research Centre for Medical Genetics, Moscow, Russia; 6 geneXplain GmbH, Wolfenbuettel, Germany; 7 Institute of Chemical Biology and Fundamental Medicine, Novosibirsk, Russia; 8 Department of Biology, Division of Natural Sciences, University of La Verne, La Verne, CA, United States of America; 9 Bioinformatics Center, AA Kharkevich Institute for Information Transmission Problems RAS, Moscow, Russia; 10 Vavilov’s Institute for General Genetics, Moscow, Russia, Moscow, Russia; Indian Institute of Science, INDIA

## Abstract

Computational analysis of promoters is hindered by the complexity of their architecture. In less studied genomes with complex organization, false positive promoter predictions are common. Accurate identification of transcription start sites and core promoter regions remains an unsolved problem. In this paper, we present a comprehensive analysis of genomic features associated with promoters and show that probabilistic integrative algorithms-driven models allow accurate classification of DNA sequence into “promoters” and “non-promoters” even in absence of the full-length cDNA sequences. These models may be built upon the maps of the distributions of sequence polymorphisms, RNA sequencing reads on genomic DNA, methylated nucleotides, transcription factor binding sites, as well as relative frequencies of nucleotides and their combinations. Positional clustering of binding sites shows that the cells of *Oryza sativa* utilize three distinct classes of transcription factors: those that bind preferentially to the [-500,0] region (188 “promoter-specific” transcription factors), those that bind preferentially to the [0,500] region (282 “5′ UTR-specific” TFs), and 207 of the “promiscuous” transcription factors with little or no location preference with respect to TSS. For the most informative motifs, their positional preferences are conserved between dicots and monocots.

## Introduction

Core promoters are the 5' regions adjacent to the transcriptional start site (TSS) and containing binding sites for transcription factors (TFBS). Computational analysis of the eukaryotic promoters is hindered by their complex architecture [1–3]. Each gene contains one or more TSS, and, respectively, one or more promoters, which initiate transcription of a gene. Depending on species, from 30% to 60% of eukaryotic genes contain the TATA motif approximately 30 nucleotides upstream of TSS. Most commonly, TATA-containing core promoters are associated with stress-related, tissue-specific and/or highly expressed genes [4]. Broadly expressed genes frequently have TATA-less promoters with a relatively broad transcription start region (TSR) replacing pronounced TSS [3, 5]. To predict the position of the TSR, characteristic promoter initiation regions (Inr) or the downstream promoter elements (DPE) may be used [1, 2].

A majority of TSS prediction software tools use sophisticated algorithms, such as oligonucleotide content-based neural network and linear discriminant approaches, while focusing on specific sequence features of the promoter region (e.g. TATA-box or CA-motif) [6]. Genome complexity affects quality of promoter predictions: for example, presence of several tissue-specific, alternative TSSs negatively affects the prediction accuracy. For the model plant *Arabidopsis thaliana*, modern algorithms identify TATA-containing promoters with sensitivities up to 95% and specificities up to 97% [2, 4, 5, 7–10]. For *Homo sapiens* and *Oryza sativa* prediction accuracies are substantially lower [10]. In case of even less studied genomes with complex organization, false positive and false negative error rates can be large, with a spurious promoter prediction occurring once per every 700–1000 nucleotides of the genome [11].

**Fig 1:**
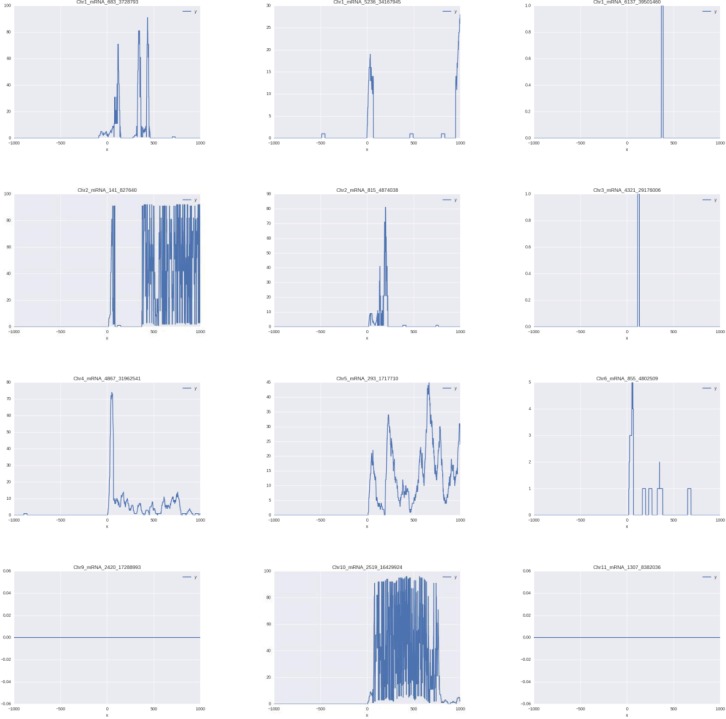
RNA-Seq coverage near 12 randomly selected promoters with experimentally validated transcription start sites.

Even the best modern methods of promoter mapping, including genomic sequencing coupled with full-length cDNA capture and ascertainment [4, 12, 13], CAGE [14, 15], 3PEAT[16], or RAMPAGE [17] are incapable to predict TSS positions with 100% accuracy [5]. For example, the mapping of CAGE tags onto existing human cDNA/mRNA sequences revealed that less than 10% of these tags fall within 10 nucleotides from TSS [18]. To illustrate this, we mapped RNA-Seq reads onto the regions TSS+/-1000 nt corresponding to 12 well-studied, experimentally validated *O*. *sativa* promoters from Plant Prom DB [3, 19], the resultant plots showed that the peaks of RNA-Seq coverage did not match the positions of known TSS, supporting the idea that correct mapping of eukaryotic promoters possibly requires multiple sources of data (Fig 1).

As me mentioned above, promoters contain transcription factor binding sites (TFBS) regulating transcription. Two most commonly used techniques to predict eukaryotic promoter by distribution of TFBS were proposed in 1995 by Kondrakhin and Kel [20] and by Prestridge [21]. The method of Kondrakhin and Kel [20] pairs up the detection of TATA boxes with the distribution of computed weight matrices of TFBS, improving the prediction accuracy compared to using the TATA box alone. Prestridge [21] combined density ratios of all individual TFBSs into a scoring profile, which was further augmented by the weighted TATA matrix. This approach reported a relatively low false positive rate. Real-world applicability of both tools, however, remains limited due to lack of species-specific TFBS models for training and failure to pinpoint locations of individual TSSs.

In the last decade, several improvements in the promoter prediction process were made. Troukhan [22] combined positional frequency of 5′ EST matches onto genomic DNA with the gene models. This approach, known as TSSer, is, in a nutshell, a deterministic method that predicts one transcription start site per locus. For *Arabidopsis thaliana* promoters, it achieves remarkable accuracy. However, even the most reliable prediction of a single promoter per gene cannot adequately reflect biological complexity underlying its regulation due to common occurrence of alternative promoters, which are often tissue-specific or responsive to the changes in architecture of chromatin [23]. In 2013, the TSSer approach was improved by incorporation of a non-parametric maximum likelihood approach to be reborn as NPEST algorithm [5], that allows prediction positions of alternative TSSs in the *A*. *thaliana* genome with better accuracy than the sequences identified in the several “gold standard” databases, such as TAIR [24, 25], Plant Prom DB [19] and Plant Promoter Database [26]. For example, for the set of 15,875 *Arabidopsis* promoters derived by both TAIR and NPEST, 11,304 (71%) were predicted within 50 nucleotides of each other, and 7,192 (45%) within 10 nucleotides of each other. Thirty percent of TAIR-predicted and 44% of NPEST-predicted promoters identified the “TATA” sequence within the interval [–40, –20] nucleotides upstream from the respective TSS. At the TSS, nucleotide consensus scores (46% of T and 49% of C followed by 65% of A) were stronger for NPEST then for TAIR (43% of T and 35% of C followed by 53% of A). When NPEST predictions were compared to experimentally confirmed promoters from other databases, similar patterns of nucleotide consensus were observed.

Recently, many more types of experimental and computational observations highlighting the TSS positions became available. For example, forty million single nucleotide polymorphisms (SNPs) from the 3,000 Rice Genomes Project (http://snp-seek.irri.org), the largest and the most dense SNP collection for higher plants [27], were shared to facilitate an analysis of genetic variants across the *Oryza sativa* cultivars [28]. Observed clusters of reduced nucleotide variability were shown to highlight functionally important genomic regions. Interestingly, a sharp decline in SNP density was noted about 250 nucleotides upstream of TSS elements; this decline reaches its minimum exactly at the TSS.

In plant genes with multiple promoters, precise mapping of TSSs requires incorporation of diverse data types including tissue/stress specificity of each transcript. Unfortunately, most of currently available techniques cannot incorporate a variety of available data, and also must ignore alternative promoters. Therefore, accurate identification of TSS and core promoter regions remains an open problem. Since evidences for location of TSS are imprecise, the best approach for promoter production should embed probabilistic integrative algorithms. In this paper, we present a comprehensive analysis of genomic features associated with the promoters and show that probabilistic integrative algorithms-driven models allow accurate classification of DNA sequence into “promoters” and “non-promoters” even in absence of full-length cDNA sequences. These models may be built upon the maps of the distributions of SNPs, RNA sequencing reads on genomic DNA, methylated nucleotides, TFBS as well as relative frequencies of nucleotides and their combinations.

## Results

### Selection of the “gold standard” gene prediction models

**Fig 2:**
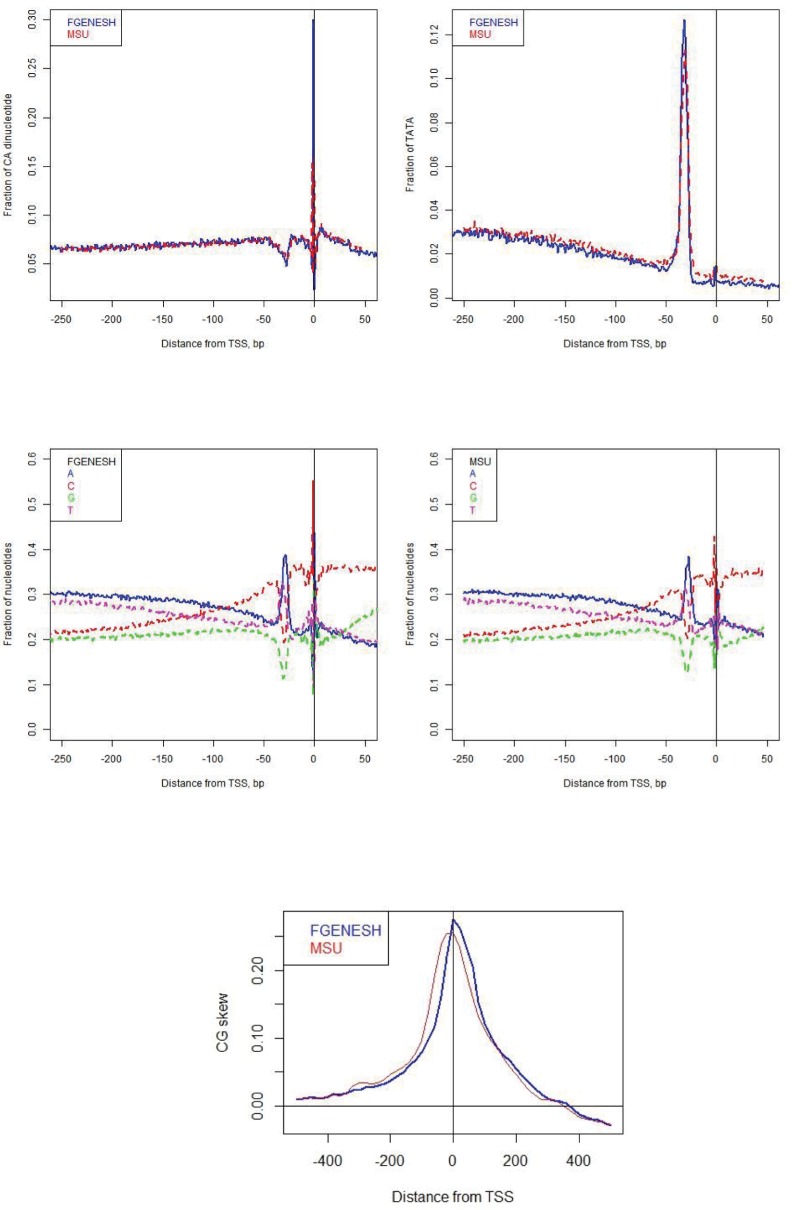
Features of the nucleotide consensus around TSS. A top left) Frequency of CA, B top right) Frequency of TATA motif, D middle feft) Frequencies of nucleotides A, C, G, T around TSS for Fgenesh, E middle right) Frequencies of A, C, G, T around TSS for MSU, F bottom) CG skew (${CG}_{skew}=\frac{\#C-\#G}{\#C+\#G}$), calculated in the window of 40 nt.

To aid a selection of the best available rice genome annotation, Fgenesh and MSU mRNA-based gene prediction models were compared. Fgenesh gene prediction set contains 18,389 high quality (5′ full, with mRNA support) gene models, while the MSU gene prediction set contains 20,367 high quality gene models [28, 29]. For every gene in both models, we extracted a 1,000 nt long sequence centered at the TSS, and calculated distributions of genomic features previously associated with the start of transcription: (1) frequency of dinucleotide CA [1, 30, 31]; (2) frequency of TATA [1, 4, 32]; (3) nucleotide consensus around TSS [12, 13, 33]; (4) CG skew (${CG}_{skew}=\frac{\#C-\#G}{\#C+\#G}$ where #C and #G refers to the counts of nucleotides C and G in a certain genomic window) [34]. Fig 2C and 2D shows that Fgenesh-annotated promoters have a more pronounced nucleotide consensus as compared to the promoters annotated by MSU. Fgenesh promoters also have higher frequency of the exact TATA motif at -30 (B), and more CA dinucleotides at the position of TSS (A). Fig 2(F) shows peak of the CG skew at TSS, calculated in the window of 40 nt both annotations; Fgenesh-annotated CG skew peak is higher than the MSU one. Based on the assumption that these features reliable reflect the quality of promoter annotation, for further analysis the Fgenesh model was selected.

### Distribution of transcription factor binding sites

The distributions of the transcription factor binding sites (TFBS) in promoters and the UTRs of high-confidence rice genes in the regions of -1000 +1000 around TSS were investigated with MATCH algorithm [35] incorporated in geneXplain platform (www.genexplain.com). MATCH uses the TRANSFAC database [36] comprising 764 plant position weight matrices (PWM) with a strict similarity score threshold of 0.95. MATCH scans the targets promoter sequences with a sliding window equal to the length of the PWM and calculates a score for each of the windows. The maximum value of the score (1.0) corresponds to the sequence that fully fits the consensus of the given PWM. Score threshold of 0.95 allows very little mismatches to the consensus, with few permitted mismatches limited to less conserved positions. In addition, the MATCH score considers the nucleotide position-specific entropy measures. In a recent study, MATCH performed with accuracy superior to other motif-finding algorithms [37].

In the Fgenesh-predicted rice promoters, MATCH search against the TRASNFAC database resulted in mapping of 3.2 million potential TFBS corresponding to 667 plant PWMs, while 97 PWMs remained matchless, possibly due to their exclusive role in the dicots or to the binding to distal promoters not analyzed in the present study (see S2 Table). Interestingly, 487 out of 667 TFBS (73%) were found in proximal promoters of *Oryza sativa* more than 1000 times; the most frequent sites were that for the transcription factors ASR1, DOF56 and PBF. When the frequencies of TFBS found in the proximal promoters were compared with the frequencies for the same PWMs found in randomly shuffled sequences, the most significant promoter-specific TFBS enrichments (twice or more) were observed for SPL12, SPL5, GBF1, ABI5, BZIP68, LEC2, and GT1 transcription factors.

**Fig 3:**
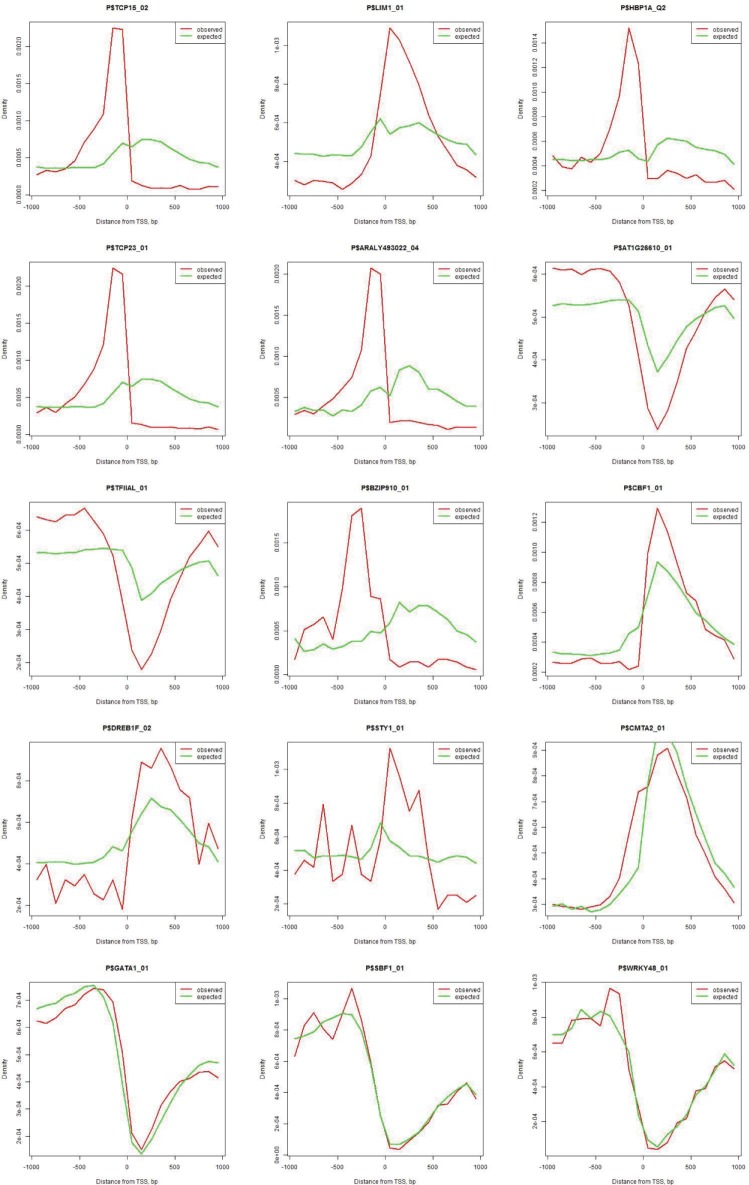
Examples of observed and expected occurrences of TFBS in rice promoters. Different: TCP15, LIM1, HBP1A, TCP23, ARALY493022, AT1G26610, TFIIAL, BZIP910, CBF1, DREB1F, STY1. Observations agree with expectations: CMTA2, GATA1, SBF1, WRKY48.

To account for dinucleotide statistics matching that of the Fgenesh rice promoter regions, another set of randomly shuffled sequences was generated as described by Stepanova, Tiazhelova [38]. Briefly, the 2000 nt regions [TSS-1000, TSS+1000] were divided onto non-overlapping 100 nt windows, then the dinucleotide statistics were calculated for each window. For each promoter, a 2000 nt long sequence with matching dinucleotide composition was generated and subjected to MATCH prediction of TFBS [35]. After selecting the motifs that occur at least in 100 different rice promoters, Kolmogorov-Smirnov test was applied to find significantly over-represented sequence motifs (S3 Table). Fig 3 shows examples of TFBS that occur at frequencies that differ and do not differ significantly between real and simulated sequences. The most pronounced differences (p-value < 0.002) were detected for the distributions of binding sites for TCP15, LIM1, HBP1A, and TCP23. On the other hand, occurrences of binding sites for CMTA2, GATA1, SBF1, and WRKY48 in real and simulated sequences were not different (p-value> 0.99999).

### Positional specificity of TFBS distribution

A phenomenon of the positional preference in TF binding was previously described by Weirauch, Yang [39], who showed that positions of TFBS are not randomly distributed in respect to the start of transcription (TSS); this observation holds across evolutionary kingdoms. To illustrate this phenomenon in rice, we divided the [TSS-1000, TSS+1000] regions into 100 nt long bins and calculated frequency histograms of TFBS occurrence in each bin; then we used *K*-means algorithm to cluster these histograms, with value at each bin treated as a separate dimension.

**Fig 4:**
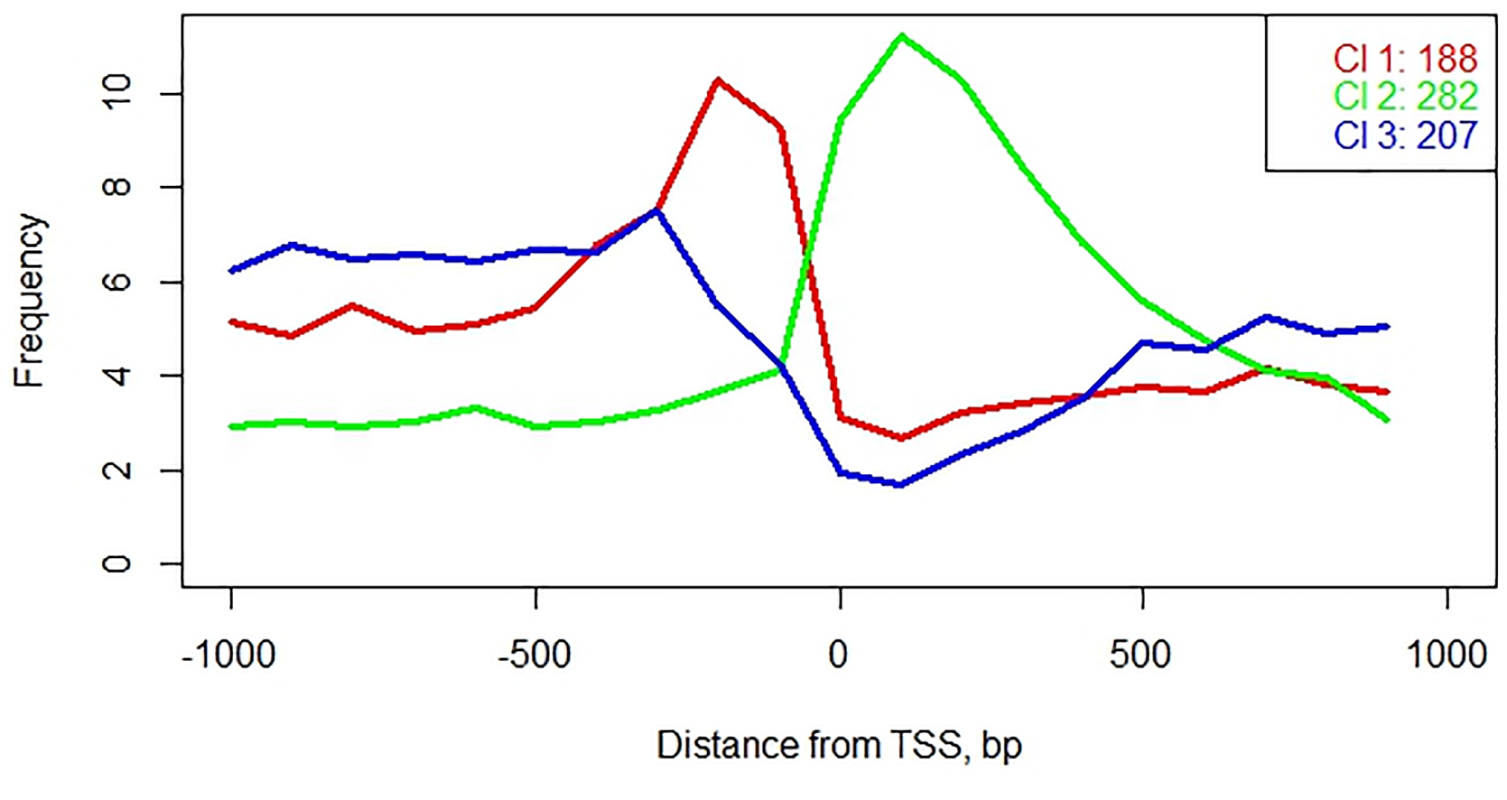
Positional specificity of TFBS distribution.

**Fig 5:**
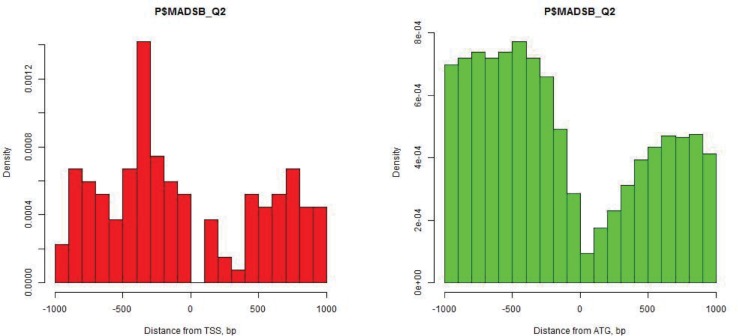
The distribution pattern for MADSB binding sites highlight the start codon (ATG) rather than the respective TSS.

**Fig 6:**
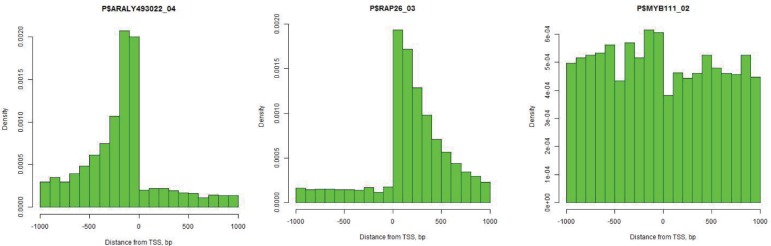
Frequency distributions of TFBS may have different patterns around the start of transcription (position 0 on the horizontal axis). X-axis shows the distance from TSS, Y-axis reflects the frequency of motif in each window. Frequencies of ARALY493022_04 TFBS (Class 1) are plotted on the left panel, of RAP26_03 TFBS (Class 2) on the middle panel, and of MYB111_02 (Class 3) on the right panel.

Positional clustering of TFBS demonstrates that the cells of *Oryza sativa* utilize three distinct classes of transcription factors: Class 1, which binds preferentially to the [-500,0] region (“promoter-specific”, N = 188); Class 2, which binds preferentially to the [0,500] region (“5′ UTR-specific”, N = 282); and Class 3, which includes predominantly “promiscuous” transcription factors with weak or no location preference for respective TSS (N = 207), see S4 Table and Fig 4. Note that some Class 3 TFs cannot be classified as promiscuous (Fig 5) as they are characterized by regular patterns of positional distribution. around the translation start rather than around the transcription start. Examples of the position frequency preference are shown in the Fig 6.

To conduct the comparative gene ontology analysis of Class 1, 2 and 3 transcription factors (Table 1), the chi-square “Goodness of Fit” tests were used: $\chi_{df=2}^{2}=\sum_{i=1,2,3}\frac{{\left(O_{i}-E_{i}\right)}^{2}}{E_{i}}$, where O_i_ and E_i_ correspond to observed and expected numbers of genes in i^th^ category.

**Table 1:** GO categories that are significantly different between three TF classes.

GO	Class 1	Class 2	Class 3	P-value
**sequence-specific DNA binding**	43	22	61	7.78E-06
**protein dimerization activity**	21	4	18	0.000438
**systemic acquired resistance, salicylic acid mediated signaling pathway**	15	2	6	0.000504
**regulation of transcription from RNA polymerase II promoter**	5	2	16	0.000558
**response to bacterium**	14	3	4	0.000955
**jasmonic acid mediated signaling pathway**	15	4	5	0.001897
**carpel development**	6	1	13	0.002606
**protein binding**	51	31	35	0.002904
**negative regulation of defense response**	11	1	5	0.003011
**DNA binding**	90	146	85	0.007339
**protein targeting to membrane**	14	4	7	0.010643
**regulation of plant-type hypersensitive response**	14	4	7	0.010643
**ethylene-activated signaling pathway**	10	20	4	0.012518
**response to water deprivation**	48	76	38	0.016138
**plant ovule development**	12	4	15	0.017373
**Nucleus**	76	126	75	0.017489
**response to ozone**	9	4	14	0.033868
**cellular response to nitrogen levels**	13	11	23	0.044478

Total number of genes with GO categories for Class 1, 2 and 3 were 130, 164, and 144, respectively. P-values were calculated using the chi-square “Goodness of Fit” procedure.

Class 1 TFs of the rice are enriched in the following GO terms: “sequence-specific DNA binding”, “protein dimerization activity”, “systemic acquired resistance, salicylic acid mediated signaling pathway”, “regulation of transcription from RNA polymerase II promoter”, “response to bacterium”, “jasmonic acid mediated signaling pathway”, “carpel development”, “protein binding”, “negative regulation of defense response”, “protein targeting to membrane”, “regulation of plant-type hypersensitive response”, “plant ovule development”, “response to ozone”. Class 2 TFs are enriched in GO terms “DNA binding”, “ethylene-activated signaling pathway”, “response to water deprivation”. Class 3 TFs are enriched in “cellular response to nitrogen levels”.

To compare expression specificity of Class 1 and Class 2 transcription factors, we used the difference of proportions test (Table 2): $Z=\frac{p_{1}-p_{2}}{\sqrt{p(1-p)(\frac{1}{N_{1}}+\frac{1}{N_{2}})}}$, where $p=\frac{{p_{1}N}_{1}+p_{2}N_{2}}{N_{1}+N_{2}}$. Genes encoding the Class 1 TFs are predominantly expressed in the petals, the sepals and the embryos of plants, while mRNAs encoding Class 2 TFs are overrepresented in the roots. This may explain previous observations of significant association of TATA motifs with expression in plant roots [4, 40]: possibly, most root-specific transcription factors bind to the 5′ UTR region rather than the region upstream of TSS.

**Table 2:** Expression specificity of TF from Class 1 and 2.

Expression pattern	Class 1 (N = 99)	Class 2 (N = 134)	Z-score
Root	66	114	-3.31344
Pollen	57	60	1.93163
Carpel	72	77	2.39883
Seed	70	74	2.40454
Leaf lamina base	61	61	2.43144
Cauline leaf	64	65	2.4497
Collective leaf structure	80	87	2.65976
Petal	72	74	2.73045
Plant embryo	78	81	2.97259
Sepal	76	76	3.17706

Z-score is calculated using the difference of proportions test.

Fig 6 shows frequency profiles for TFBSs of ARALY493022_04 (Class 1, left panel), RAP26_03 (Class 2, middle panel), and MYB111_02 (Class 3, right panel). ARALY493022 is basic helix-loop-helix factor, with GGGCCC consensus sequence. Presence of GGGCCC in the region upstream of TSS is associated with the elevated level of gene expression [4, 39, 41, 42]. RAP2.6 is a defense-related, ethylene response transcription factor which recognizes the GCC-box and characterized by high affinity to DNA sequence GCGCCGCCG [43]. Ali, Abbas [44] experimentally showed that RAP2.6 works both in tissue-specific and stress-specific manner. Under normal conditions, expression of RAP26 is elevated in roots and stems, while being significantly reduced when plant is infected with pathogenic nematodes, such as *H. schachtii*. To suppress resistance responses, nematodes downregulate expression of RAP2.6 in host cells. MYB111 is involved in the regulation of several genes of the flavonoid biosynthesis pathway in cotyledons and leaves [45, 46]; it confers tolerance to UV-B [47]. Its binding site MYB111_02 has consensus G[G/T]TAGGT[A/G] [43]. MYB111 is an example of TFs with relatively weak position specificity related to TSS. The TFBS motifs occur no very often; they usually provide condition-specific regulation of genes. Fig 6 demonstrates utility of Class 1 and Class 2 TFBS for TSS prediction, while the mapping of the Class 3 TFBS does not convey additional positional information about the TSS.

According to the Kolmogorov-Smirnov test, three classes of TFs differ in the significance of over-representation of their TFBS in promoters and in the randomly shuffled sequences: thirty-seven percent of the Class 1 TFs with motifs located predominantly upstream of TSS were significantly overrepresented (p-values <0.05), In the Class 2 TFs with TFBS located in 5′ UTRs, overrepresentation was confirmed for 20% of the PWMs. The TFBS for Class 3 TFs were distributed evenly. For the latter group, significant over-representation was detected for 15% of class members (S2 Table).

In summary, three classes of TFBS differ in their position specificity, percentages of PWMS significantly over-represented in real promoters, functional classification of their downstream genes, and the patterns of their gene expression.

### Evolutionary conservation of TFBS position information content

**Fig 7:**
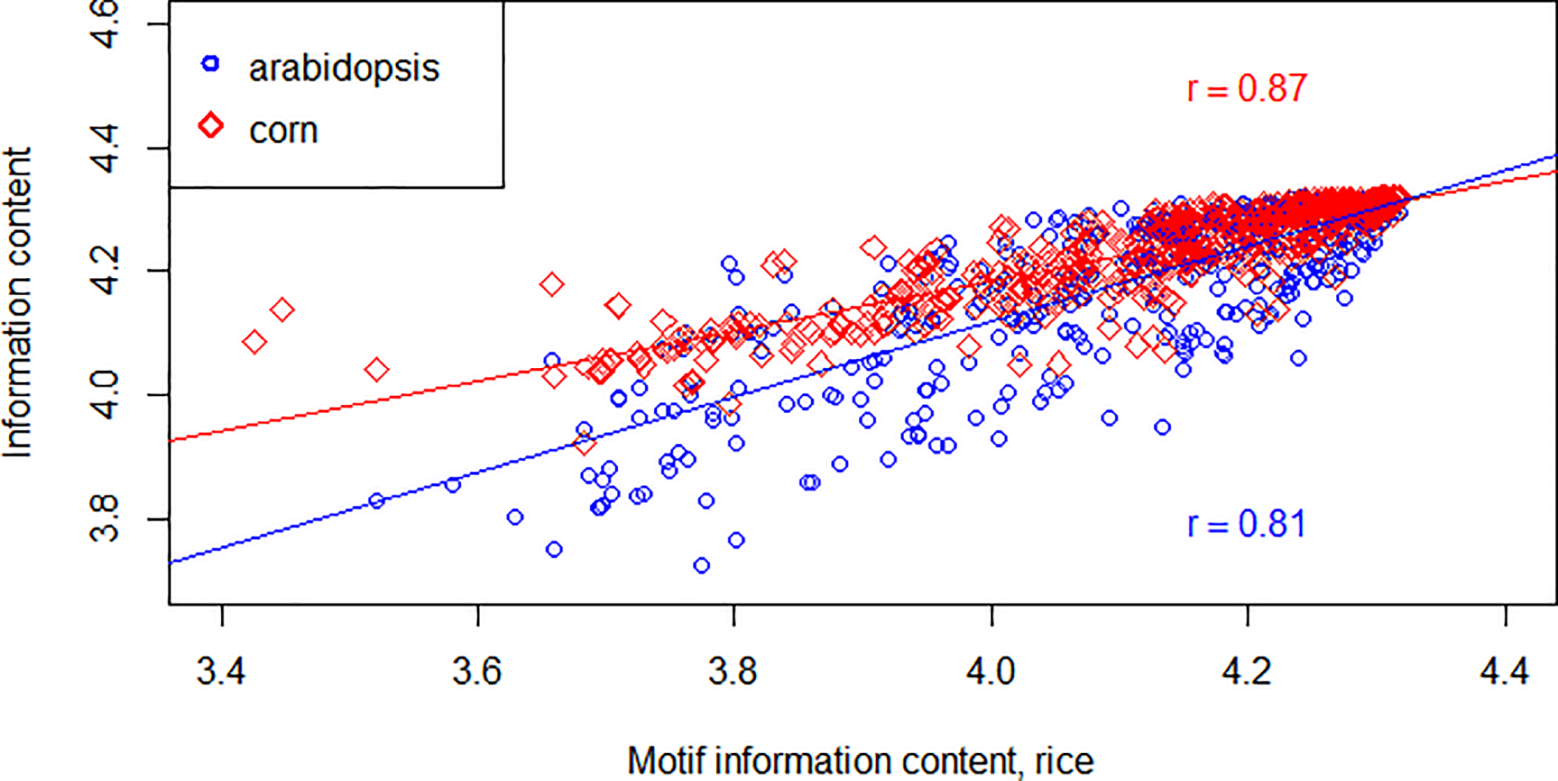
Relationship between information content of TFBS positions in rice, corn and Arabidopsis. Each point corresponds to one transcription factor; X axis shows information content in rice, Y axis–information content in corn and Arabidopsis.

We have analyzed evolutionary conservation of the TFBS position information content (a measure of unevenness of the motif distribution along promoter regions, see Method section) in monocots *Oryza sativa* and *Zea mays*, and in the dicot *Arabidopsis thaliana* (Fig 7). Following correlations between these measures were identified:

$$I_{corn}=2.575339+0.402007{\times I}_{rice}$$

Multiple R^2^ = 0.7504, Adjusted R^2^ = 0.75, F-statistic: 1819 on 1 and 605 DF, p-value: < 2.2E-16

$$I_{arabidopsis}=1.69125+0.60706\times I_{rice}$$

Multiple R^2^ = 0.6512, Adjusted R^2^ = 0.6506, F-statistic: 1083 on 1 and 590 DF, p-value: < 2.2E-16.

**Fig 8:**
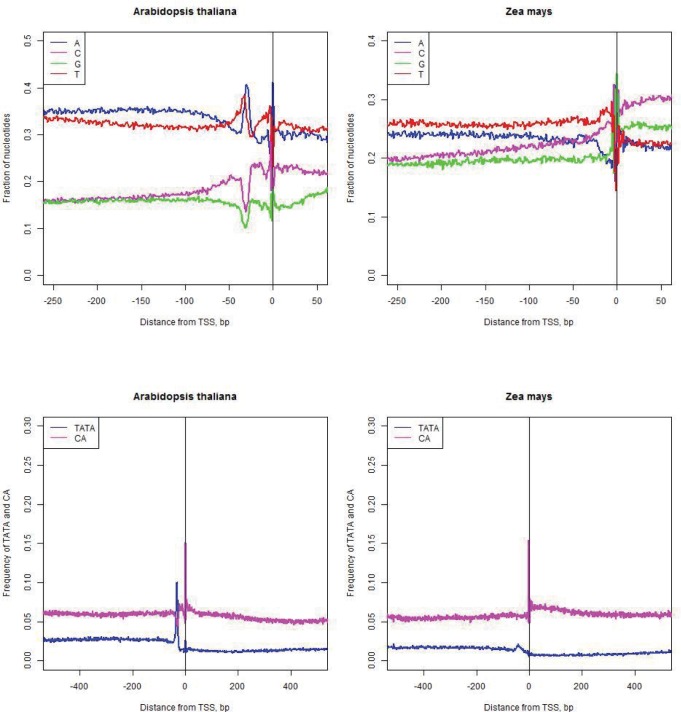
Assessment of promoter prediction quality in Arabidopsis (left) and corn (right). Arabidopsis genome shows more pronounced consensus at TSS, with higher frequency of TATA motif at -30 and CA at TSS.

Correlation of the TFBS position information content in two monocots (rice and corn) were higher than that for the rice and a dicot plant Arabidopsis. By extracting TFBS with more than 10,000 matches in each of three plant genomes, a list of 46 “common” informative TFBS was compiled. Each of these TFBS was classified into either “promoter-specific” or “5′ UTR-specific” category in each species. Between rice and corn, 42 of 46 “common” TFBS are consistent in their position preference (see S7 Table). Between rice and arabidopsis, the agreement is seemingly higher, with 45 of 46 TFBS of the common set having the same positional preference (see Supplemental Data). We hypothesize that this discrepancy is due to lower reliability of the TSS map in corn genome as compared to arabidopsis and rice genomes (Fig 8). Importantly, this phenomenon may lead to a systematic “shifting” of the TFBS peaks from promoters to 5′ UTRs and vice versa. Fortunately, incorrect prediction of TSS in corn does not affect the information content of a TFBS, and correlation coefficient of motif information content between two grasses (rice and corn) is 0.87, which is above the correlation between rice and arabidopsis (Fig 7). In summary, positional preference of the most informative motifs remains conserved between dicots and monocots.

### Identification of similar TFBS

**Fig 9:**
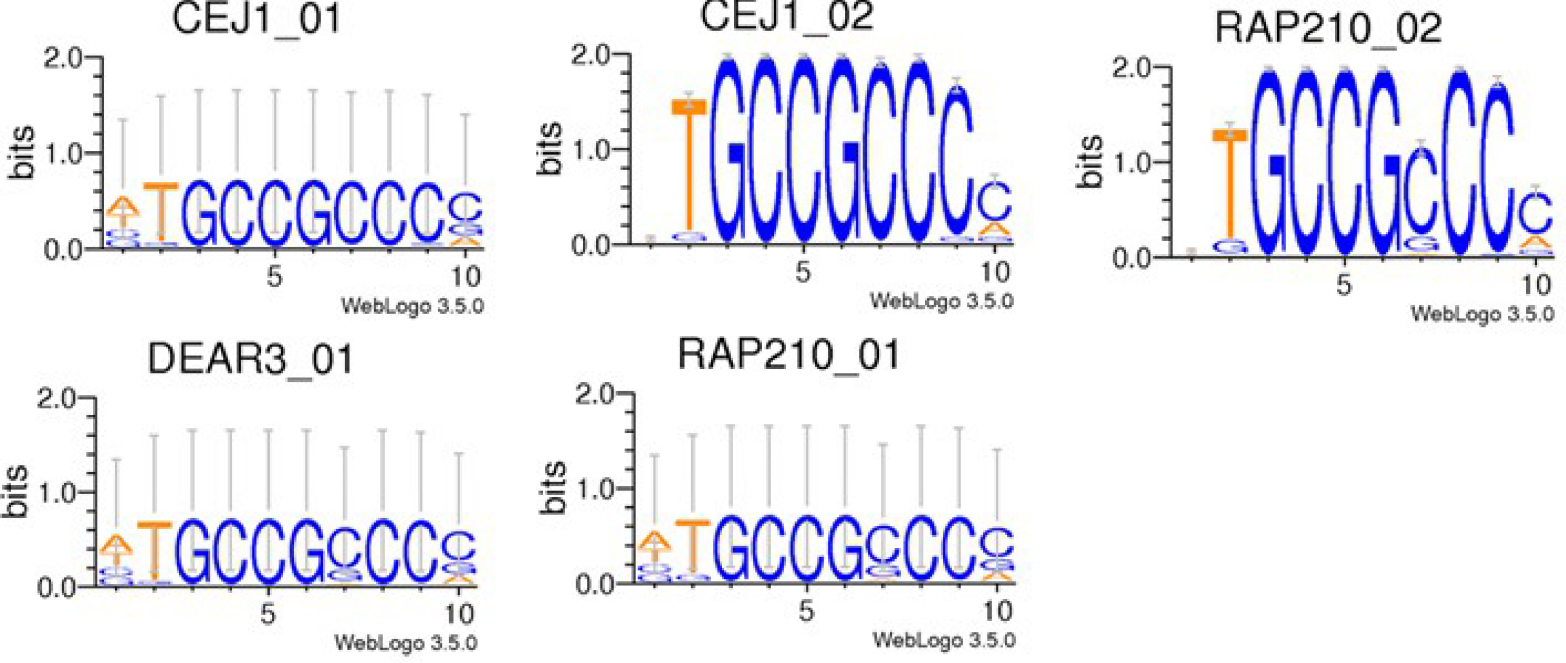
An example of five distinct TFBS entries in the TRANSFAC database with very similar position weight matrices (PWMs).

Since TRANSFAC database tends to accumulate all published motifs, some of collected motifs appear to be redundant. For example, several PWMs may be independently built and reported for the same transcription factor (Fig 9). Also, transcription factors of the same protein family may recognize highly similar motifs, which will be reflected by similarities of respective PWMs. Fig 9 shows TFBS logo plots for a group of transcription factors with highly similar motifs. Although regulatory functions of these may vary, for a practical use in promoter prediction, these motifs should be clustered into a non-redundant set based on similarity of their PWMs. By clustering 764 plant PWMs, a non-redundant set of 376 sequence motifs was obtained, among which, forty-six were found informative with the scores above 0.0138 (see S1 Table).

### Nucleotide variants resulting in the TFBS loss and gain

**Fig 10:**
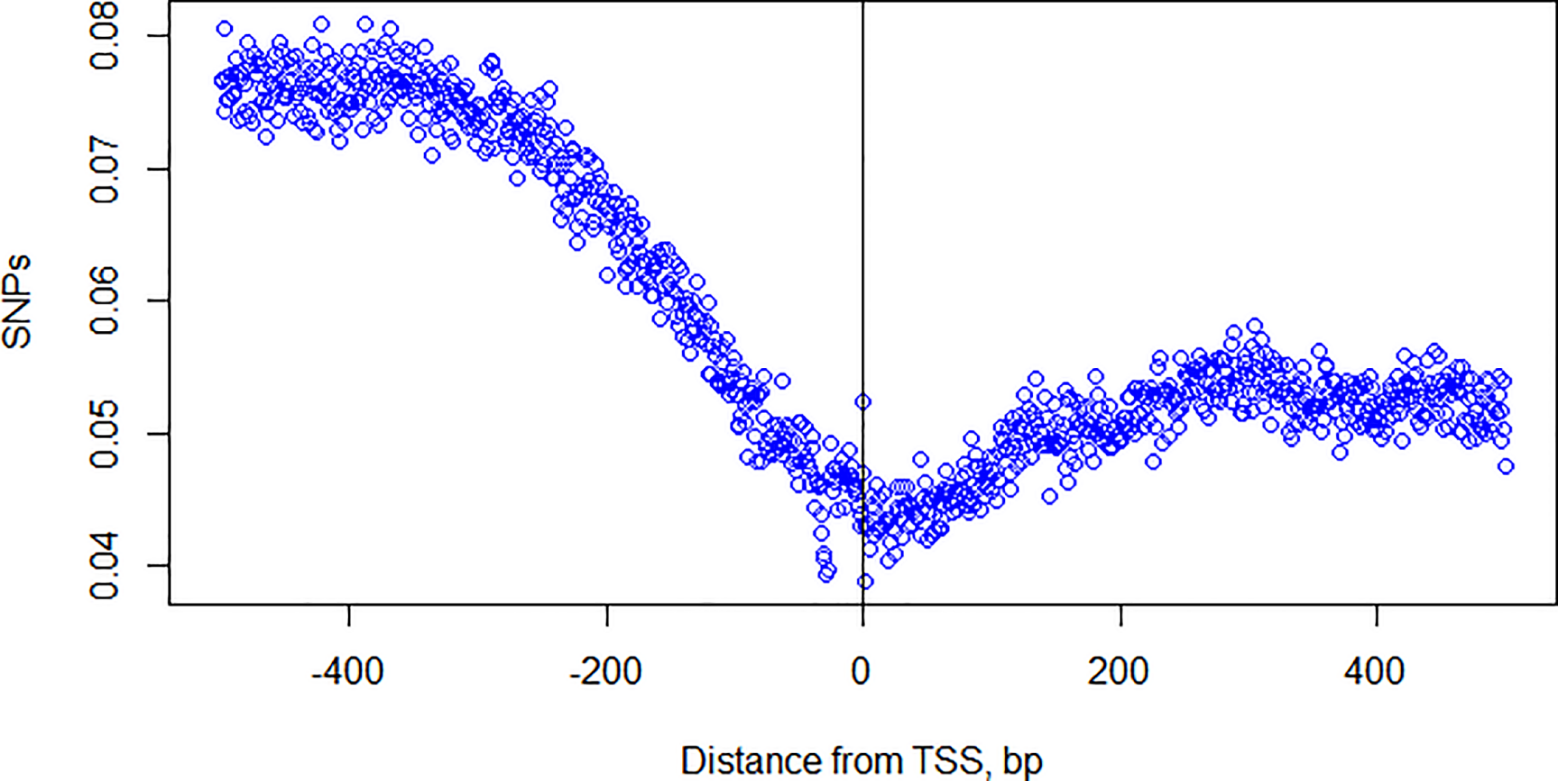
Frequency of SNPs located near the TSS in rice.

Core promoters and 5′ UTR regions located within 200 bp around the TSS are both protected against accumulation of nucleotide variants (Fig 10). This protective effect is due to selection constraints, which prevents disruption on regulatory elements located near TSS by neutral or near-neutral genomic variants. Cross-analysis of comprehensive collection of plant TFBS [37] and an extensive dataset of the genomic variants detected in various rice cultivars [27] allowed us to classify regulatory elements of these plants according to their tolerance to the mutations (see S5 Table).

To achieve that, we considered distribution of SNPs and their effects on loss and gain of TFBS. For each nucleotide change, we have calculated Δ = |*q* − q*| for the TFBS scores before (q) and after (q*) nucleotide change, and compared its values to empirically determined thresholds. Calculations of the scores q and q* were done according to the MATCH scoring formula (see Materials and Methods). If Δ ≥ Δ_0_, the site was considered as “lost” or “gained” depending which score value was larger, q or q*.

Frequencies of site losses and site gains for the promoters and for the random subset of 18,389 intergenic sequences, each 2,000 nt in length, were compared. We hypothesized that functionally important promoter motifs will have less variation causing the loss of sites. For each TF, we calculated the ratio of the site losses in intergenic sequences to the site losses in promoters. All entries were than ranked according to these ratios, which reflected relative “suppression” of the site losses by SNPs (Table 3). Relative suppressions of the site gains were calculated in a similar fashion (see Table 4). The binding sites for ABF (CACGTGGC) and CBF4 transcription factors were the most protected from the site loss. In abscisic acid signaling, ABF factors govern osmotic stress response through modulation of the gene expression downstream of SnRK2 kinases, while CBF4 regulates adaptation to drought. For several important transcription factors, such as MADS8 (involved in the control of flowering time), GT-1 and GATA-1 (response to light), we observed that variation was avoided in positions where nucleotide change can lead to the site gain. Additional data and the results of the analysis of SNPs in TFBS could be seen in the Table 3, Table 4, and S5 Table.

**Table 3:** Suppression of site loss caused by nucleotide variants in promoters.

ID	Frequency intergenic/ Frequency promoters	#Promoter Sites	#Intergenic Sites	P-values
P$AT2G20350_01	1.856	1909	3660	6.15E-112
P$ARF1_01	1.814	856	1604	3.30E-47
P$DREBIII4_01	1.677	870	1507	2.78E-35
P$AT2G41690_01	1.648	1072	1825	5.37E-40
P$CBF1_03	1.540	2803	4459	8.89E-74
P$DREB1F_01	1.534	4873	7720	4.52E-124
P$ORA47_01	1.529	4129	6521	8.35E-104
P$RAP210_01	1.527	4519	7128	1.11E-112
P$DEAR3_01	1.526	4525	7134	1.51E-112
P$RAP210_02	1.526	4525	7134	1.51E-112
P$ERF019_01	1.526	4199	6620	1.36E-104
P$RAP21_01	1.526	4236	6675	3.17E-105
P$AT1G71520_01	1.525	4242	6682	3.57E-105
P$DREB1B_01	1.449	2544	3808	1.16E-48
P$HSF3_01	1.423	1262	1855	1.08E-22
P$AT4G16610_01	1.374	8852	12563	7.08E-118
P$AT4G16750_01	1.347	1175	1635	2.62E-15
P$AT2G44940_01	1.338	1231	1701	2.99E-15
P$MADS17_01	1.307	7134	9628	1.37E-66

“Frequency intergenic”/“Frequency promoters” is the ratio between frequencies of site loss due to SNPs located in intergenic regions and the site loss due to SNPs located in promoters.

**Table 4:** Suppression of site gain caused by nucleotide variation in promoters.

ID	Frequency intergenic/ Frequency promoters	#Promoter Sites	#Intergenic Sites	P-values
P$WRKY23_01	1.376	1691	2403	2.59E-24
P$FUS3_Q2	1.331	2249	3091	2.07E-25
P$BHLH112_01	1.319	1813	2471	1.14E-19
P$MYB46_02	1.290	1015	1352	4.37E-10
P$WRKY_Q2	1.286	10925	14507	1.56E-88
P$TGA2_Q2	1.275	6140	8084	3.08E-47
P$CDC5_01	1.269	3653	4787	8.44E-28
P$MADS4_01	1.266	1948	2548	1.89E-15

Column “Frequency intergenic”/“Frequency promoters” contains the ratio of the frequency of site gain due to SNPs located in intergenic regions to the frequency of the site gain due to SNPs located in promoters.

The binding sites for AT2G20350 and ARF1 transcription factors were the most “protected” from the site loss. AT2G20350 factors regulate activity of ethylene-activated signaling pathway. The plant hormone ethylene is involved in many aspects of the plant life cycle, including seed germination, root hair development, root nodulation, flower senescence, abscission, and fruit ripening (Johnson and Ecker, 1998). ARF1 is a member of the auxin response factor family, involved in hyperosmotic salinity response. For several important transcription factors, such as WRKY23 (involved in hyperosmotic salinity response and response to auxin), FUS3 (plays a role in embryonic development ending in seed dormancy and response to auxin stimulus), we observed that variation was avoided in positions where nucleotide change can lead to the site gain.

### Distribution of RNA-Seq reads

**Fig 11:**
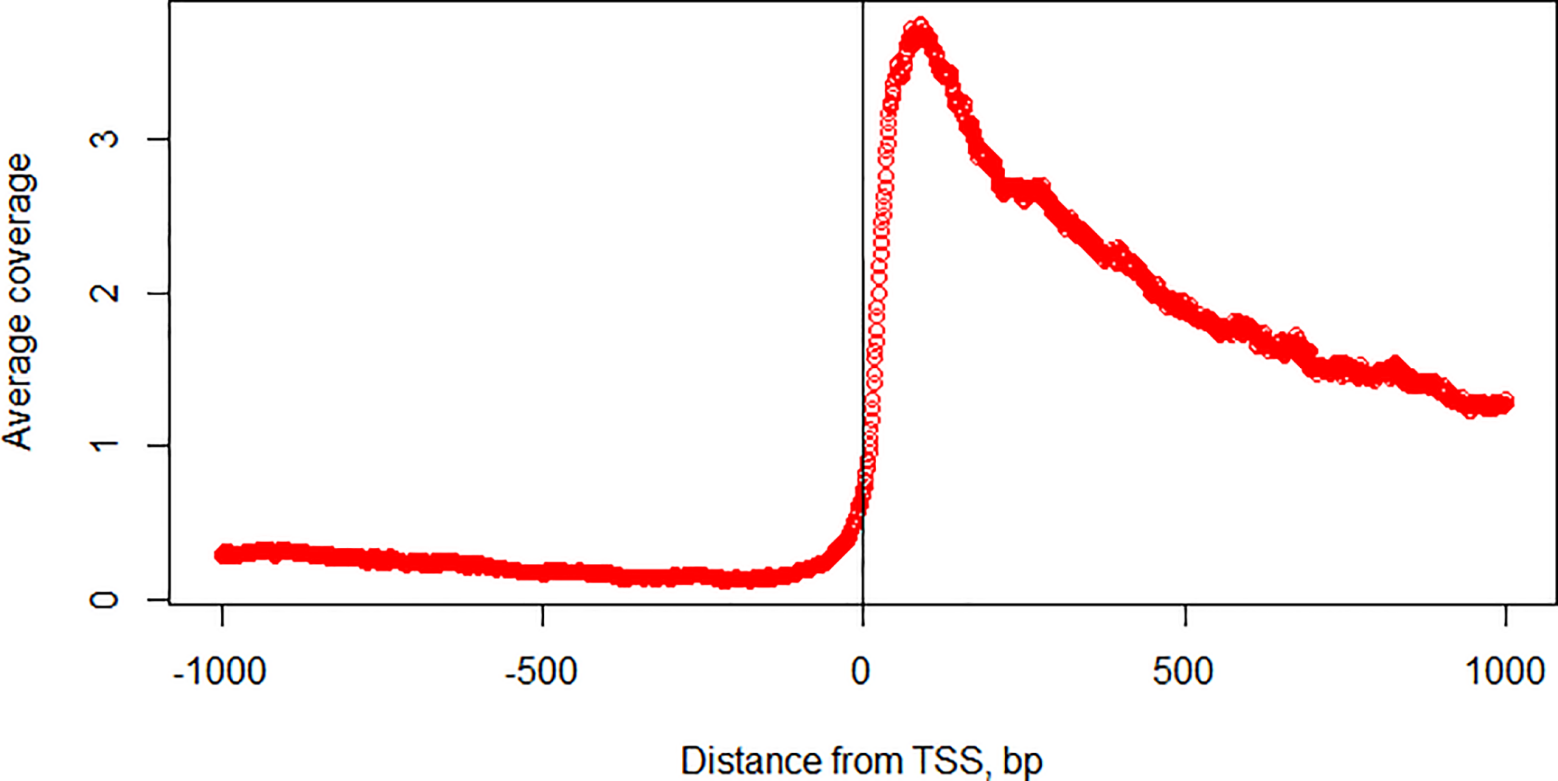
RNA-Seq coverage near the transcription start site.

Predictably, an analysis of mapped RNA-Seq reads near TSS [-1000; +1000] showed that, on average, coverage peaks are observed immediately downstream of TSS (Fig 11). However, some genes lack a peak of RNS-Seq reads at their TSS. Notably, only 26% of rice genes display a maximum of the coverage in the range [-50, +250], and only 60% of genes display this maximum in the range [-50, +550].

### R-loop forming sequences (RLFS)

Three-stranded nucleic acid R-loop structure is formed between nascent RNA transcript and DNA template [48]. Length of the R-loop sequence varies between 150 to 650 nt. R-loops aid in the prevention of methylation within promoters [49–51] and are associated with initiation of transcription and other important gene-level features [48]. In particular, R-loops accumulate at the G-rich 5′-UTR regions immediately downstream of the CpG-non-methylated human promoters [50]. To map the R-loop forming structures in the area [TSS-1000, TSS+1000], we used the QmRRFS tool [48, 52, 53]. QmRRFS partitions R-loops into three segments, the RIZ (DNA region of initiation of R-loops containing at least three contiguous guanines), the linker (a spacer up to 50 nt between RIZ and REZ), and the REZ (G-rich region supporting extension of R-loop, up to 2000 nt long). In agreement with Ginno, Lott [50], QmRRFS-driven analysis showed that 22% of rice genes are associated with at least one R-loop in the area [TSS-1000, TSS+1000], with the predominant localization in 5′-UTR. The observed distribution of RLFS was unimodal, with the peak of the distribution located at the position around 200 nt downstream from the TSS; over a half of RLFS (52%) were found in the 5′-UTR [TSS, TSS+400] (S6 Table). Notable, this peak coincides with the region where polymerase typically pauses after the initiation of transcription [48, 52, 53].

### DNA methylation

**Fig 12:**
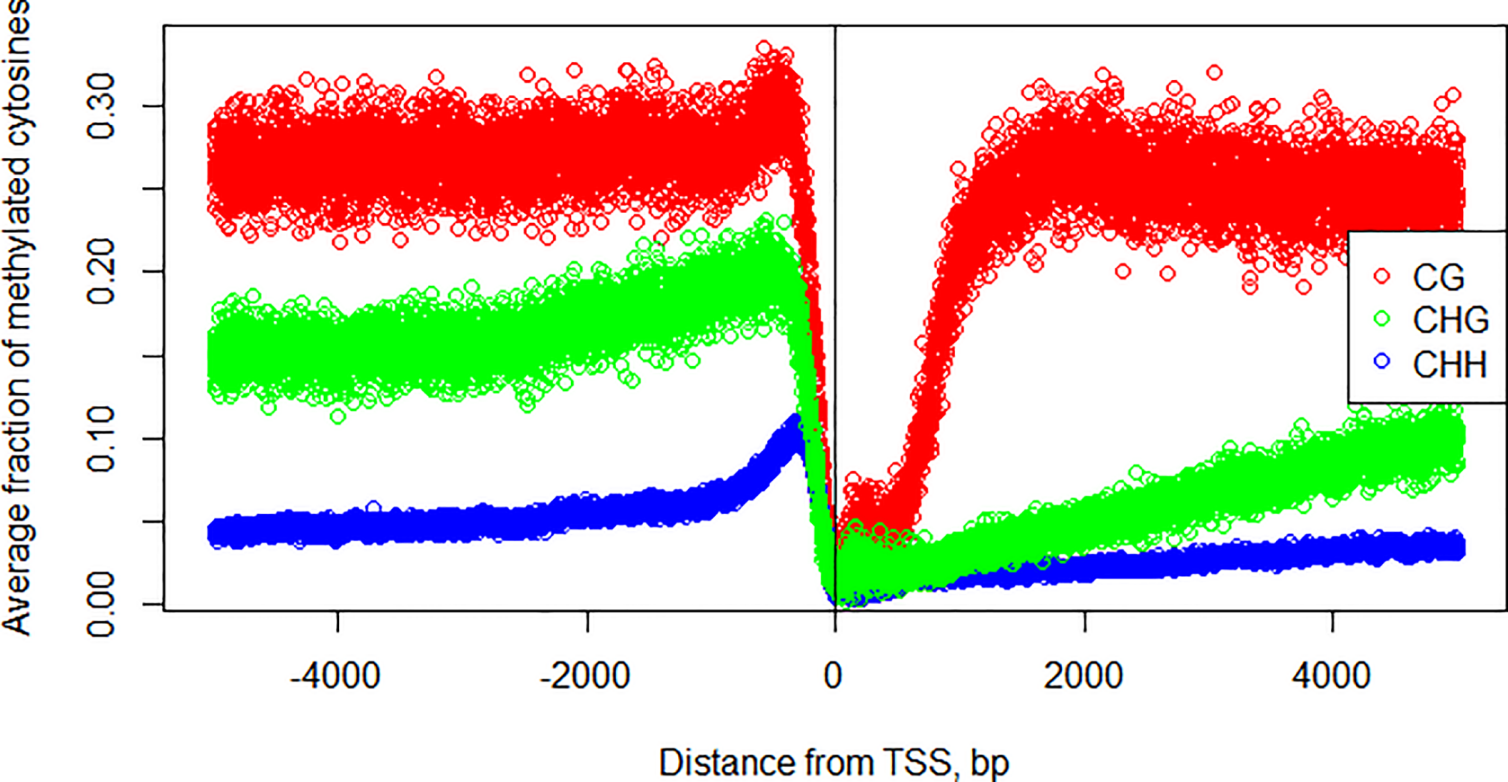
Methylation around transcription start site in rice in different sequence contexts. Red–CG, green—CHG, blue–CHH, where H denotes A, C or T nucleotide.

In the intergenic regions and within functional classes of genes and their promoters, the patterns of DNA methylation predictably differ [54, 55]. The most pronounced effect was observed for the methylated CpGs (see Fig 12). Intergenic level of CpG methylation was at 0.27, with sharp decline starting around 600 bp upstream of TSS to about 50% of that in intergenic region level at the position of -170, then proceeds to its minimum (0.01) at 8 bp upstream from the TSS.

### Combining the characteristic features of TSS into promoter classifier

We used 18,389 “promoter” (positives) and 18,389 “non-promoter” (negatives) sequences. To train the model, we used 14,711 positives and negatives; and the remaining 3,678 positives and negatives were used for testing. The binary classifier interrogates the candidate sequence and reports whether the sequence is “promoter” or “non-promoter”. The best combination of features was: composition of DNA sequence, GC-skew value and presence/absence of the CA-motif in every position. It achieved the best accuracy (0.9995) and has the Matthews correlation coefficient of 0.9989 (see Table 5). Other features also improve the classification accuracy in comparison with the DNA sequence alone, however, not performing as well as the combination of DNA sequence, GC-skew and CA-motif distribution.

**Table 5:** Promoter classification accuracy.

Features	TP	TN	FP	FN	Accuracy	Sensitivity	Specificity	CC
DNA sequence	3424	3030	648	254	0. 8774	0. 9309	0. 8238	0.7591
DNA sequence + CG skew	3635	3653	25	43	0.9907	0.9883	0.9932	0.9832
DNA sequence + CG skew + frequency of CA motif	3674	3678	0	4	0.9994	0.9989	1.0	0.9989
DNA sequence + CG skew + RNA-Seq coverage	3658	3666	12	20	0.9956	0.9945	0.9967	0.9913
DNA sequence + CG skew + frequency of TATA motif	3653	3608	70	25	0.9870	0.9932	0.9810	0.9742
DNA sequence + CG skew + DNA methylation	3657	3563	115	21	0.9815	0.9942	0.9687	0.9633
DNA sequence + all TFBS	3241	3386	292	437	0.9009	0.8812	0.9206	0.8024
DNA sequence + all TFBS +CG skew	3619	3668	10	59	0.9906	0.9839	0.9973	0.9813
DNA sequence + selected TFBS+CG skew	3628	3674	4	50	0.9927	0.9864	0.9989	0.9854
DNA sequence + SNP	3430	3138	540	248	0.8929	0.9326	0.8532	0.7882
DNA sequence + SNP+CG skew	3348	3296	382	330	0.9032	0.9103	0.8962	0.8065
DNA sequence + CG skew+ RNA-Seq coverage +selected TFBS	3653	3663	15	25	0.9946	0.9932	0.9959	0.9891
DNA sequence + CG skew + frequency of CA motif + RNA-Seq	3665	3638	40	13	0.9928	0.9965	0.9891	0.9856

## Discussion

In this work, we have investigated several features of promoter area, identified characteristic patterns of their distribution and assessed utility of these features for identification of TSS location. Accuracy of TSS identification affects the overall quality of regulatory region analysis. To date, large amounts of the “mapped” TSS are, in fact, defined only approximately. A significant fraction of promoters has multiple alternative TSS, many of which are not yet annotated. These features make prediction of exact positions of TSS a very complex problem. Further work toward exact mapping of all TSS positions using various promoter characteristics in multiple species is warranted. It is essential to find and annotate tissue- and condition-specific transcription start sites and associate them with alternative splice form, gene regulatory network, and protein function.

Intelligent integration of multiple types of genomic information (DNA composition, regulatory elements, DNA methylation, RNA-Seq coverage data, SNP distribution etc.) may improve annotation of tissue- and developmental stage-specific genes that are often misidentified due to their atypical sequence composition in grasses [54–56]. We showed that the region containing promoter-UTR boundaries could be defined using the following pronounced trends: (1) drop in SNP density, (2) evolutionary conserved peaks and valleys of the positions of regulatory elements, (3) peak of RNA-Seq coverage immediately downstream from the TSS, (4) peak of CG skew, (5) drop in DNA methylation density in CpG, CHH and CHG contexts, where H denotes A, C or T nucleotide. Integration of multiple noisy features of promoter regions can result in 99% classification accuracy. Features identified as important by deep learning based classification can now be used to build a scoring function for promoter prediction.

In our work, we focused on the 2000 nucleotide long region around rice TSS, supported by experimental evidence. In rice, the median length of 5′ UTR is 120 nt; with less than 1.2% of 5′ UTRs being larger than 1000 nt [28]. Therefore, for the vast majority of loci, the considered regions covered both transcription and translation start sites, being sufficient for description and classification of rice promoters.

Analysis of SNPs in the context of TFBS in promoter and non-promoter region indicated that TFBS differ by their tolerance to nucleotide variation. It is of note that the binding sites for AT2G20350 and ARF1 transcription factors were the most “protected” from the site loss. Both of these factors are involved in plant hormone signaling [57]. We conclude that sites for these transcription factors are “protected” in evolution from being lost due to their importance for regulation of plant lifecycle. It was interesting to observe that for several transcription factors nucleotide variations were avoided in positions where nucleotide change can lead to the site gain. Among such factors were WRKY23 and FUS3, involved in gene regulation in response to the plant hormone auxin. We propose that spurious generation of novel sites for these transcription factors may significantly alter cellular timing. We conclude that TRANSFAC analysis may results in functional observations as it provided clear evidence of interplay between SNPs and TF binding sites in rice genome.

## Materials and methods

### Fgenesh++ rice gene prediction

Fgenesh++ (Find genes using Hidden Markov Models) [58–60] is a HMM-based *ab initio* gene prediction program [61]. We used the rice chromosomes (version MSU 7, [29]) to make the initial gene prediction set, applying the Fgenesh gene finder with generic parameters for monocot plants. From this set, we selected a subset of predicted genes that encode highly homologous proteins (using BLAST with E-value cut-off 1.0E-10) to known plant proteins from the NCBI non-redundant (NR) database. Based on this subset, we computed gene-finding parameters, optimized for the rice genome, and executed the Fgenesh++ pipeline to annotate the genes in the genomic scaffolds. The Fgenesh++ pipeline used all available supporting data, such as known transcripts and homologous protein sequences. NR plant and, specifically, rice transcripts were mapped to the rice genomic sequences, therefore identifying a set of potential splice sites. Plant proteins were mapped to the rice genomic contigs, and the high scoring matches were selected to generate protein-supported gene predictions, so that only the highly homologous proteins were used in gene identification.

Amino acid sequences from predicted rice genes were then compared to the protein sequences from plant NR database using the 'bl2seq' routine, and the similarity was significant if it had a BLAST percent identity ≥ 50, BLAST score ≥ 100, coverage of predicted protein ≥ 80% and coverage of homologous protein ≥ 80%. BLAST analysis of the predicted sequences was also carried out against the *O*. *sativa* mRNA dataset, using an identify cutoff of >90%. Predictions that have both NR plant RefSeq and *O*. *sativa* mRNA support, as well as the 5′ UTR longer than 20 nucleotides and shorter than 1000 were selected for the analysis.

GFF file with Fgenesh++ gene prediction is available as a Supplemental Data file.

### MSU rice gene models

The current MSUv7 annotation (http://rice.plantbiology.msu.edu) of rice genome contains 55,986 predicted genes and 66,338 gene models [29]. Upon exclusion of pseudogenes, transposable elements, and genes with atypical lengths of 5′ UTR (below 20 nt or above 1000 nt long), a high-confidence set contains 20,367 expressed protein-coding rice genes.

### Arabidopsis gene and promoter models

Genome annotation files for TAIR 10 version and sequences for 3000 nucleotides upstream from ATG were obtained from The Arabidopsis Information Resource (TAIR) [24, 62]. The upstream sequences were truncated based on the position of the nearest upstream locus. 290,085 EST sequences were obtained from NCBI and TAIR and mapped onto the 27,199 upstream sequences using nucleotide BLAST + (minimum identity percent: 95%; maximum query start of alignment: 5; only plus strand alignments were used). Using the text search, we removed ESTs annotated as 3′ or partial. NPEST [5] algorithm was used and resulted in prediction of 17,452 transcription start sites for 16,520 protein-coding loci.

### Corn gene and promoter models

Genome annotation of maize (B73, 6a) contains 40,602 predicted protein-coding genes [63]. We excluded genes with atypical lengths of 5′ UTR (below 20 nt or above 1000 nt long), genes without full-length mRNA support, without valid start and stop codon, or no PFAM annotation. This filtering resulted in 16,180 putative corn TSS.

### Positional information content of transcription factor binding sites

We selected TFBS that occur at least 10,000 times in promoters of a given species. In rice, it amounted to 487, in Arabidopsis -559, and in corn—171 TFBS. To calculate the information content of each TFBS, we divided the region around the start of transcription (TSS-1000, TSS+1000) into 100 nt long bins, and calculated the observed frequency of TFBS matches in every window as a ratio of matches within the window to the total number of matches $f_{o}=\frac{m}{T}$. The expected frequency is calculated as $f_{e}=\frac{1}{Number\;of\;windows}$. The information content (a.k.a. Shannon’s entropy) is defined as $I=\sum_{windows}f_{e}log(\frac{f_{e}}{f_{0}})$. The binding sites were ranked from highest to lowest information content.

### RNA-Seq data

We used following publicly available rice RNA-Seq datasets: SRR034580, SRR034581, SRR034582, SRR034583, SRR034584, SRR034585, SRR034586, SRR034587, SRR034588, SRR034589, SRR034590, SRR034591, SRR034592, SRR034593, SRR034594, SRR034595, SRR034596, SRR034597, SRR034598, SRR034599, SRR042529, SRR074125, SRR074126, SRR074127, SRR074128, SRR074129, SRR074130, SRR074131, SRR074132, SRR074133, SRR074134, SRR074135, SRR074136, SRR074137, SRR074139, SRR074140, SRR074142, SRR074143, SRR074144, SRR074145, SRR074146, SRR074147, SRR074149, SRR074150.

The datasets were processed using the following protocol:

1. Duplicates were removed using tool *clumpify* (http://jgi.doe.gov/data-and-tools/bbtools/bb-tools-user-guide/clumpify-guide/) allowing for up to two errors per read.
2. Quality trimmed using *trimmomatic* [64] with minimum read length = 16, minimum quality 28 (sliding window of length 10)
3. Aligned to the MSU 7 rice genome using *Hisat2* [65] aligner.

Summary statistics is shown in the Table 6.

**Table 6:** RNA-Seq dataset quality.

Experiments	Reads	Read length	Quality	Aligned
SRR034580-SRR034599	~5.5 M	35	Poor	67–72%
SRR042529	8.5M	36	Good	84%
SRR074125-SRR074150	2–5M	26	Good	~1.5% (!)

### Identification of transcription factor binding sites

The prediction of TF binding sites is done using the MATCH tool, which is based on the usage of information vector-based PWM model. This model calculates the *matrix similarity score* (*q*) defined in [35]. This model is a common additive model, which uses a transformed matrix instead of an initial matrix, where each column of the transformed matrix is determined with the help of weighting the corresponding initial column by information content. The matrix similarity score *q* is calculated according to the following formula:

$$q=\frac{\sum_{i=1}^{L}I(i)f(b_{i},i)-\sum_{i=1}^{L}I(i)f^{min}(i)}{\sum_{i=1}^{L}I(i)f^{max}(i)-\sum_{i=1}^{L}I(i)f^{min}(i)}$$

here, *L* is the length of the weight matrix; *b*_*i*_ is the nucleotide that is observed in the position *i* of the sequence of TF binding site; *f(b*_*i*_,*i)* is the frequency of nucleotide *b*_*i*_ in the position *i* of the weight matrix; *f*^*min*^*(i)* is the frequency of the nucleotide which is the rarest in the weight matrix in the given matrix position *i*; *f*^*max*^*(i)* is the highest frequency the given matrix position *i*. The information content *I(i)* in the position *i* is defined as followed:

$$I(i)=\sum_{B\in \{A,C,G,T\}}f(B,i){log}_{2}(4f(B,i))$$

It describes conservation of the position *i* of the weight matrix. Multiplication of the nucleotide frequency by the information content imposes penalty on consensus mismatches in highly conserved regions of the matrix. We have recently demonstrated that this strategy is superior to the common alternative approaches of computing the TFBS scores [37].

#### Site loss and gain

We analyzed distribution of SNPs and their effect on TF binding site loss and gain. The effect of a SNP on TF binding sites was computed as the follows. For each SNP and for each PWM model we computed two matrix similarity scores (see above): *q* and q* corresponding to two nucleotides in the SNP–the reference and alternative nucleotides.

Next, we calculated Δ = |*q* − q*|, and compared its value to the empirically determined threshold Δ_0_. If Δ ≥ Δ_0_, the site was considered as “lost” or “gained” depending on sign of the difference *q* − q*.

We then calculated frequencies of site loss and site gain for all considered SNPs to identify which transcription factor binding sites (TFBS) are significantly enriched by the effect of nucleotide changes in SNPs analyzed. As a background, we considered random nucleotide changes in random genomic positions. We denote study and background sets briefly as “*Yes”* and “*No”* sets (the “*Yes”* set is the set of TFBS sequences overlapping SNPs with either the reference nucleotide or alternative nucleotide; the “*No”* set is the set created by random nucleotide substitutions in random genomic positions). The algorithm for TFBS enrichment analysis, called F-Match, has been described in Kel, Konovalova [66] and Koschmann, Bhar [67]. Briefly, the procedure finds a critical value (a threshold) for the differences between scores q and q* (*the threshold* Δ_0_) of each PWM in the library that maximizes the *“Yes/No”* ratio *R*_*YN*_ as defined in Eq (1) under the constraint of statistical significance:

RYN=#SitesYes#SitesNo#SeqYes#SeqNo (1)

In Eq (1), *#Sites* and #*Seq* are the sites and sequences counted in “*Yes”* and “*No”* sets. A high “*Yes/No”* ratio indicates strong enrichment of binding sites for a given PWM in the “*Yes”* sequences. The statistical significance is computed as follows:

$$P(X\geq x)=\sum_{n=x}^{N}(\begin{array}{c} N\\ n \end{array})p^{n}{\left(1-p\right)}^{N-n}$$ (2)

$$p=\frac{{\#Seq}_{Yes}}{{(\#Seq}_{Yes}+{\#Seq}_{No})}$$

$${N=\#Sites}_{Yes}+{\#Sites}_{No}$$

$${n=\#Sites}_{Yes}$$

The Yes/No ratio and P-value is computed separately for the site gain and for the site loss. If “*Yes/No”* ratio >1 and a P-value < 0.01 for a given PWM we consider this as an indication of an enrichment of SNPs by the sites for the given PWM. We can say that sites of this PWM are frequently effected by the SNPs and, therefore, the gene regulation by the respective TFs is significantly altered by the considered SNPs.

#### Matrix clustering

Many matrices in the TRANSFAC database are highly similar, up to the point being undistinguishable. To lower the complexity of the training data, we performed hierarchical clustering and used only one matrix from each cluster for promoter classification. The distance between two motifs is calculated as sum of squared differences between all matrix elements. If matrices were not the same size, we slide the shorter matrix over the longer one and take minimal distance. The cut-off for merging clusters was determined empirically by considering the sequence logos of matrices to be merged at each step and deciding which matrices we consider duplicates.

### Classification of promoter regions

**Fig 13:**
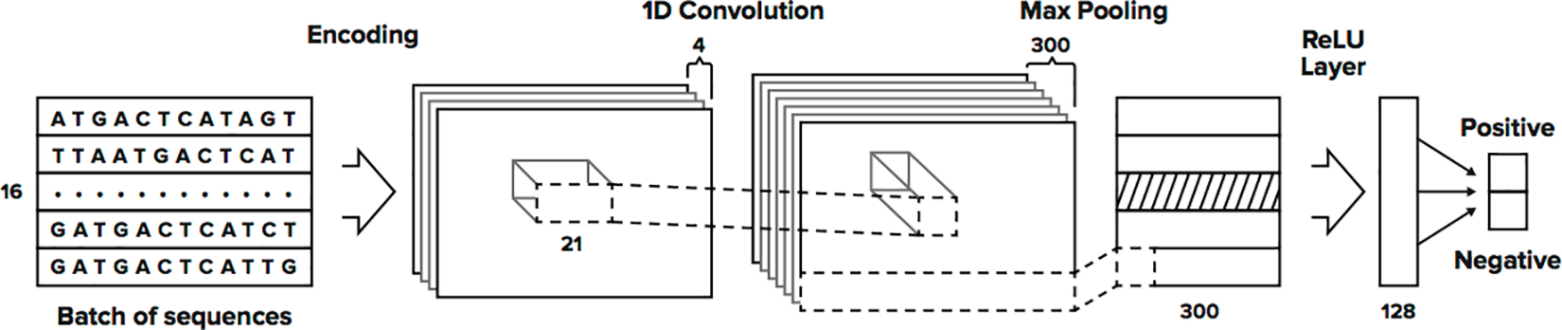
Basic CNN architecture that was used in building promoter models implemented in the learnCNN.py program [3, 10].

There are many network architectures and the task is to choose a suitable one for a given research problem. We used Convolutional Neural Networks (CNN) architecture for building promoter recognition models developed by Umarov and Solovyev [10]. The software consists of several modules. In the ***learnCNN*.*py*** modules the CNN model was implemented using *Keras*—a minimalist, highly modular neural networks library, written in Python. It uses the *Theano* library as a backend and utilizes GPU for fast neural network training. *Adam* optimizer was used for training with categorical cross-entropy as a loss function. Our CNN architecture (Fig 13) consists of one convolutional layer with 200 filters of length 21. After the convolutional layer, there is a standard Max-Pooling layer. The output from the Max-Pooling layer is fed into a standard fully connected ReLU layer with 128 neurons. Pooling size was equal to 2. The ReLU layer is connected to the output layer with sigmoid activation, where neurons correspond to promoter and non-promoter classes. The batch size used for training was 16.

Input of the network consisted of nucleotide sequences where each nucleotide is encoded by a four-dimensional vector A (1,0,0,0), T (0,1,0,0), G (0,0,1,0) and C (0,0,0,1) and other dimensions filled by other promoter features such as: GC-skew, DNA methylation, SNP, presence of CA motif, presence of TATA motifs, TFBS. The output is a two-dimensional vector: “promoter” (1, 0) and “non-promoter” (0, 1) prediction. ***learnCNN*.*py*** learns parameters of the CNN model and outputs the accuracy of promoter prediction for the test set of sequences. It also writes the computed CNN Model into a file, which can be used later in programs for promoter identification in each sequence. We used 70% of these examples for learning, 10% for validation (to find an optimal number of learning epochs) and 20% for testing.

We have extracted 18,389 sequences around transcription start site determined by full-length mRNA. Sequence [TSS-199, TSS+50], containing 200 nucleotides from promoter and 50 nucleotides from 5’ UTR, was designated as the “promoter” region, and sequence [TSS+751, TSS+1000], from the coding part of the gene, as “non-promoter”.

Quality of prediction was assessed using the following measures: True Positives (TP), True Negatives (TN), False Positive (FP), False Negative (FN), Accuracy, Sensitivity, Specificity, Matthews correlation coefficient (CC):

$$Accuracy=\frac{TP+TN}{TP+TN+FP+FN}$$

$$Sensitivity=\frac{TP}{TP+FN}$$

$$Specificity=\frac{TN}{TN+FP}$$

$$CC=\frac{TP\times TN+FP\times FN}{\sqrt{\left(TP+FP)(TP+FN)(TN+FP)(TN+FN\right)}}\text{-}$$

## Supporting information

S1 Data Annotation of *Oryza sativa* genome using the Fgenesh++ pipeline. (ZIP)

S1 Table Positional specificity and information content of plant regulatory elements. (DOCX)

S2 Table TRANSFAC plant position weight matrices. (XLSX)

S3 Table Over-representation analysis of position weight matrices. (XLSX)

S4 Table Clusters of location preference of regulatory elements in rice. (XLSX)

S5 Table Mutation tolerance of regulatory elements. (XLSX)

S6 Table Distribution of R-loop forming sequences in promoters. (XLSX)

S7 Table Positional conservation of regulatory elements between rice, corn and arabidopsis. (XLSX)
