# Supplementary material for: Nucleotide patterns aiding in prediction of eukaryotic promoters
Source: PLoS One. 2017 Nov 15;12(11):e0187243. doi: 10.1371/journal.pone.0187243 (PMC5687710; doi:10.1371/journal.pone.0187243)
Supplement: S1 Table — (DOCX) [file pone.0187243.s002.docx]

| **Motif name** | **Logo** | **Histogram** | **Information content** |
| --- | --- | --- | --- |
| ABI4_01 | 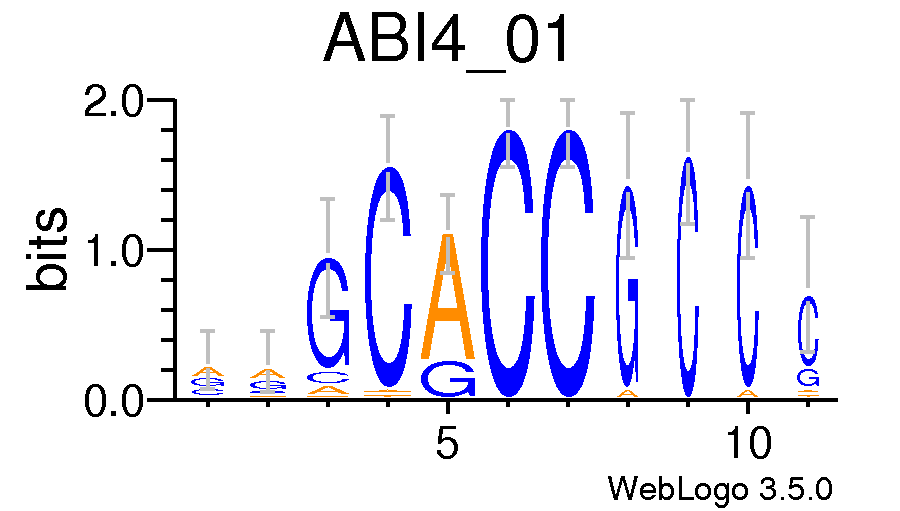 | 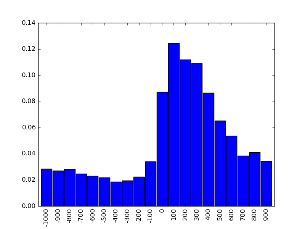 | 4.025966 |
| ABI5_01 | 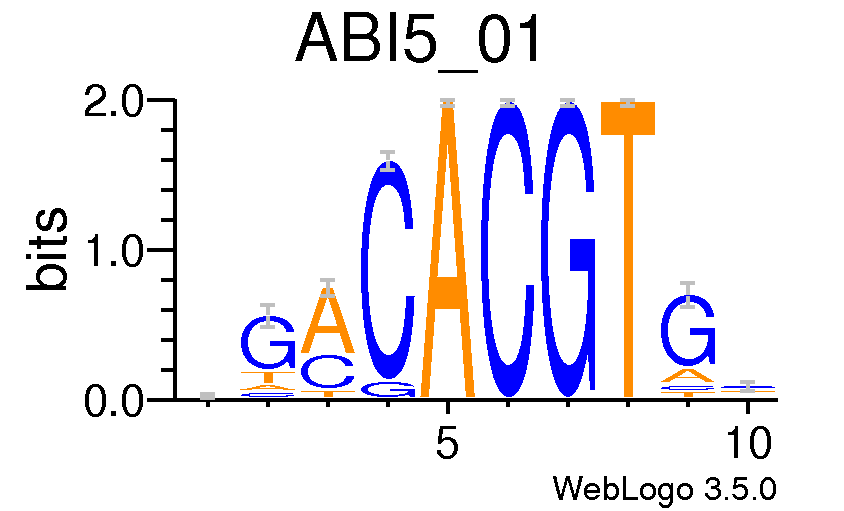 | 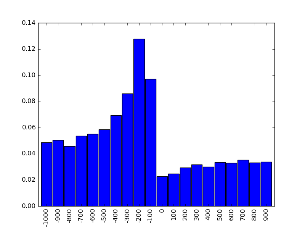 | 4.148407 |
| ABR1_01 | 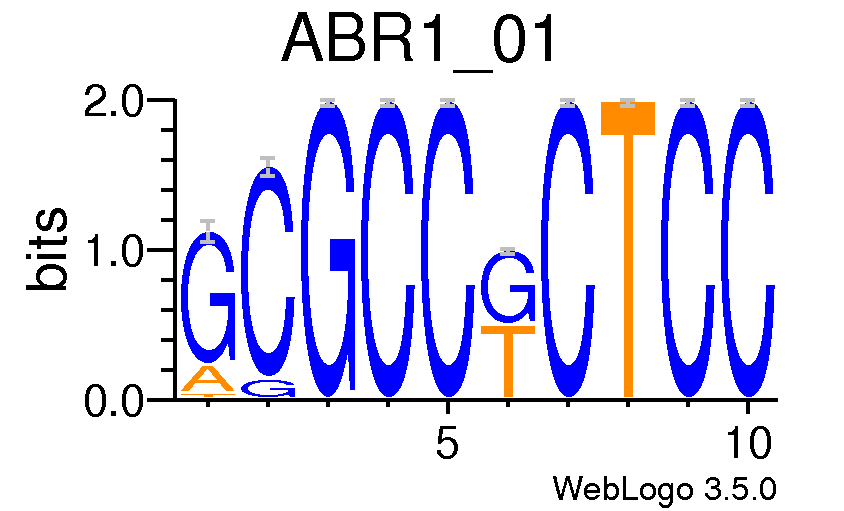 | 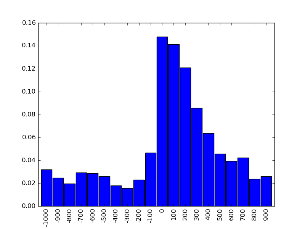 | 3.940859 |
| AHL12_01 | 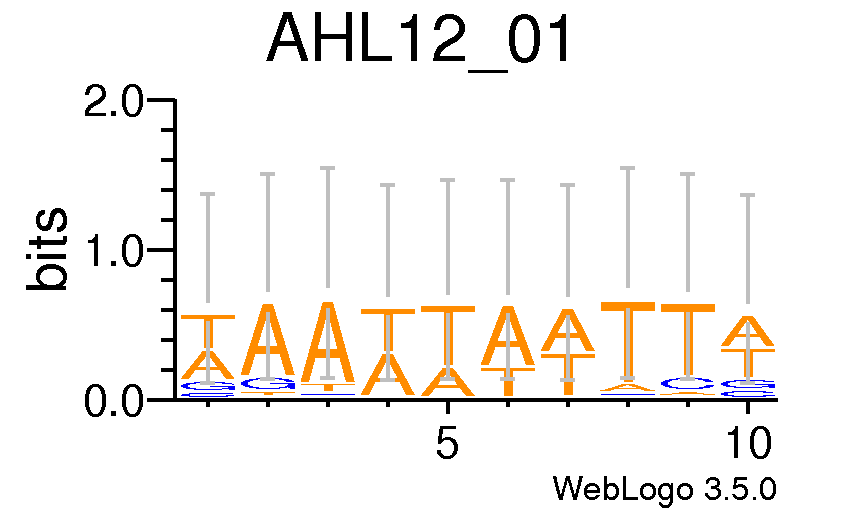 | 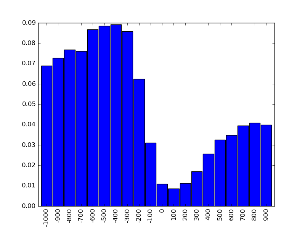 | 4.071731 |
| AHL20_01 | 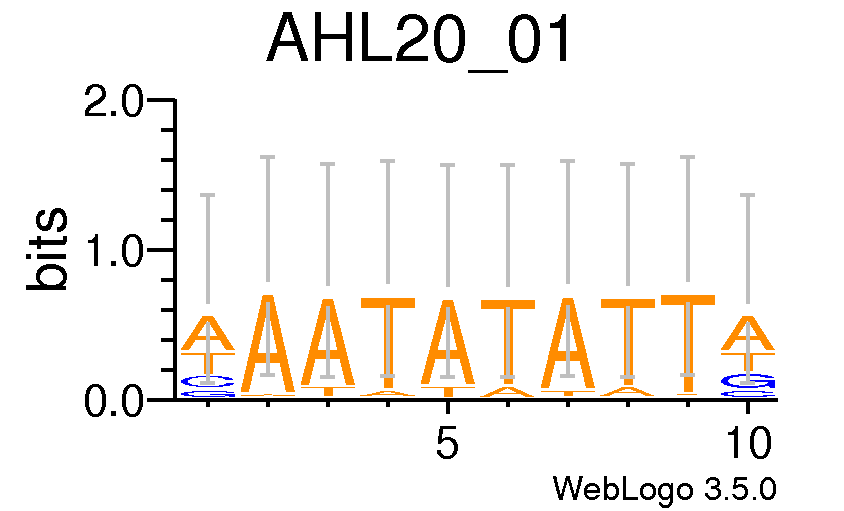 | 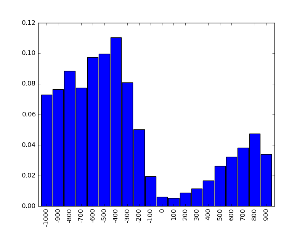 | 3.954068 |
| ALFIN1_Q2 | 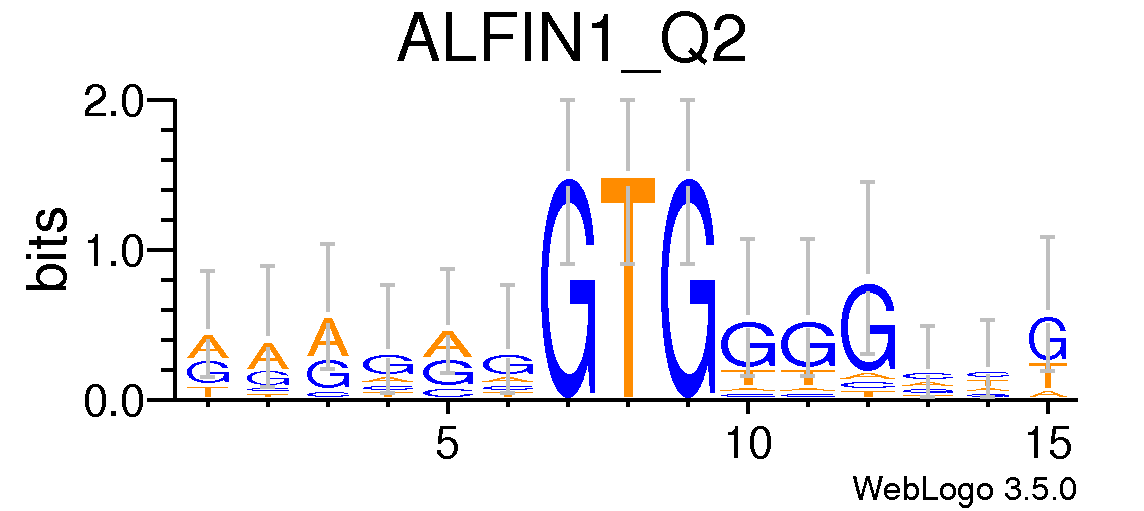 | 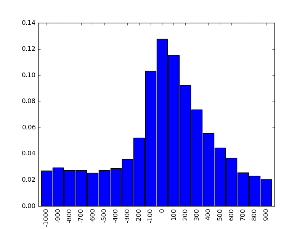 | 4.050139 |
| ARALY493022_04 | 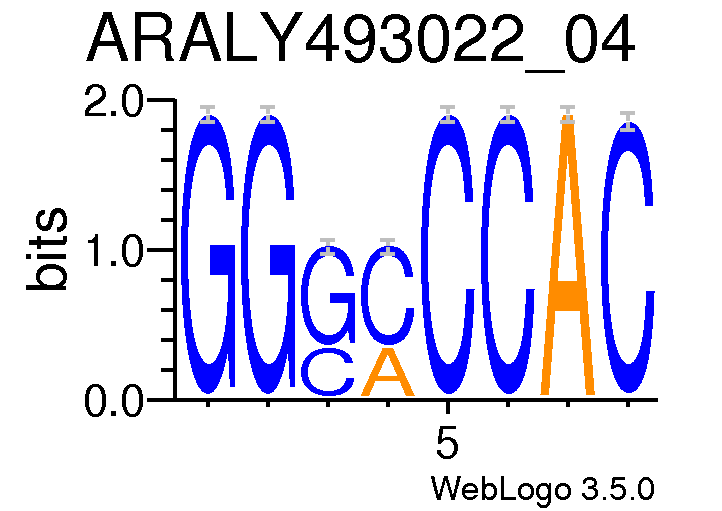 | 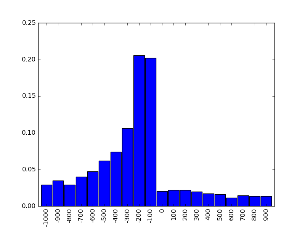 | 3.657365 |
| AT1G77950_01 | 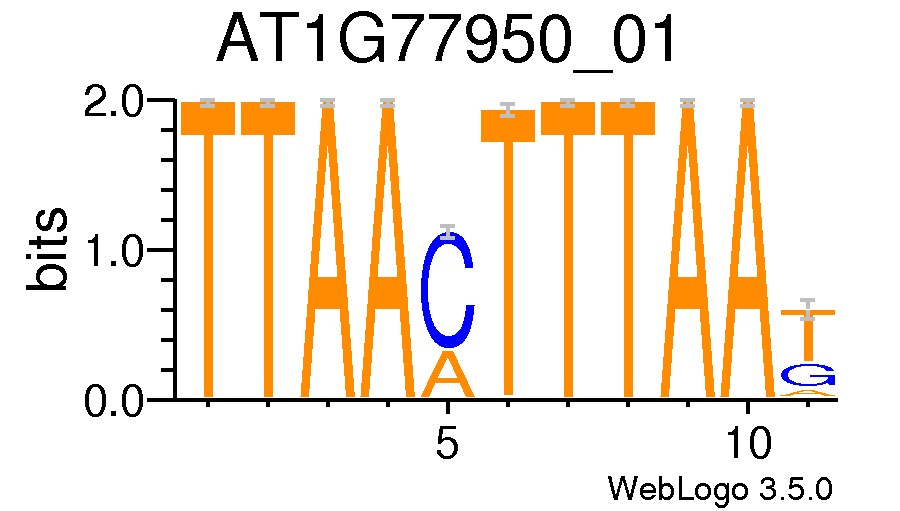 | 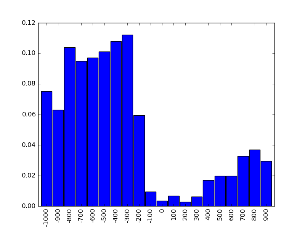 | 3.820525 |
| AT3G20750_01 | 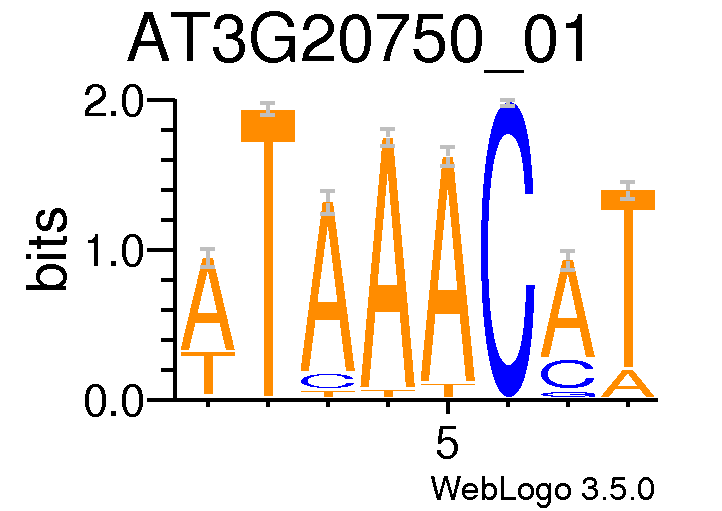 | 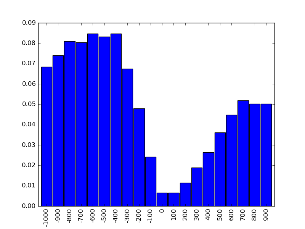 | 4.079943 |
| AT3G63350_01 | 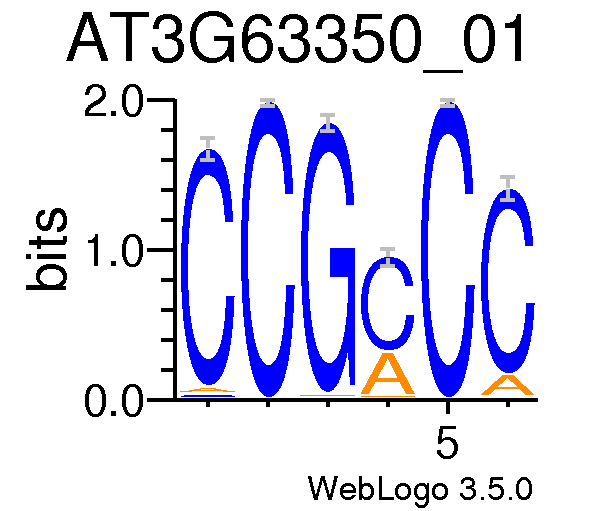 | 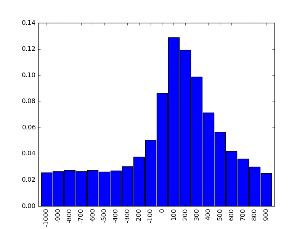 | 4.064090 |
| ATHB15_01 | 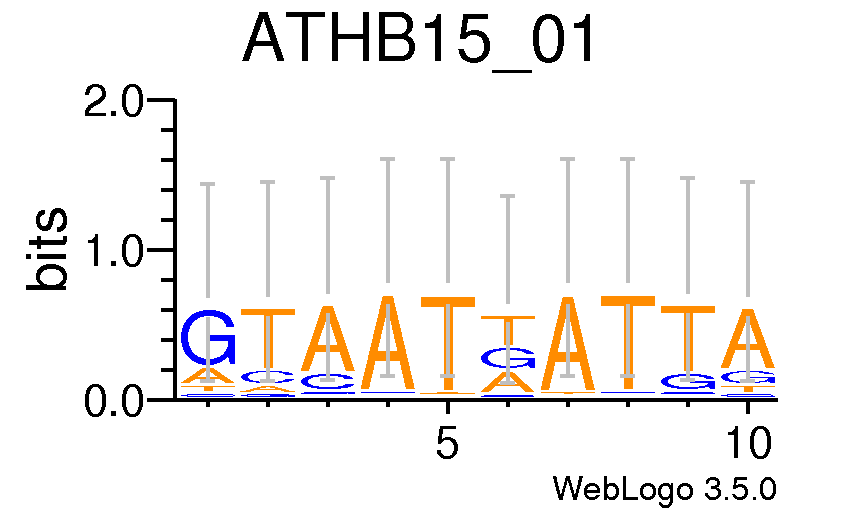 | 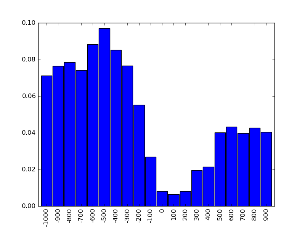 | 4.054740 |
| ATHB16_01 | 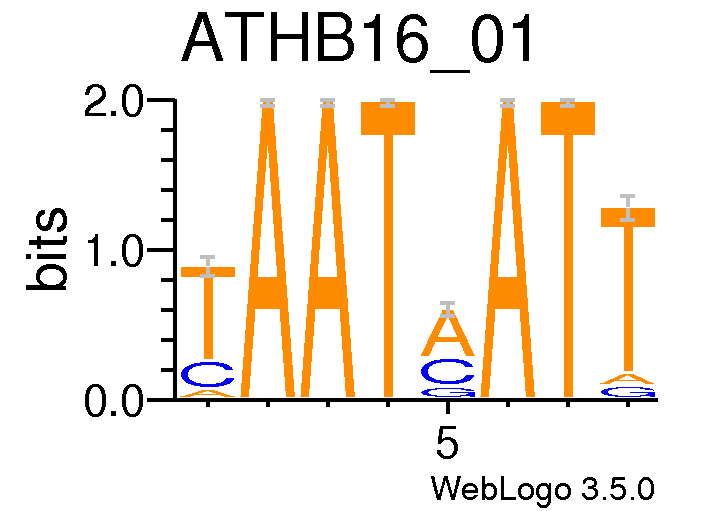 | 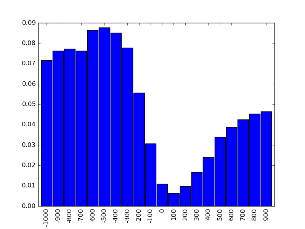 | 4.073509 |
| AZF3_01 | 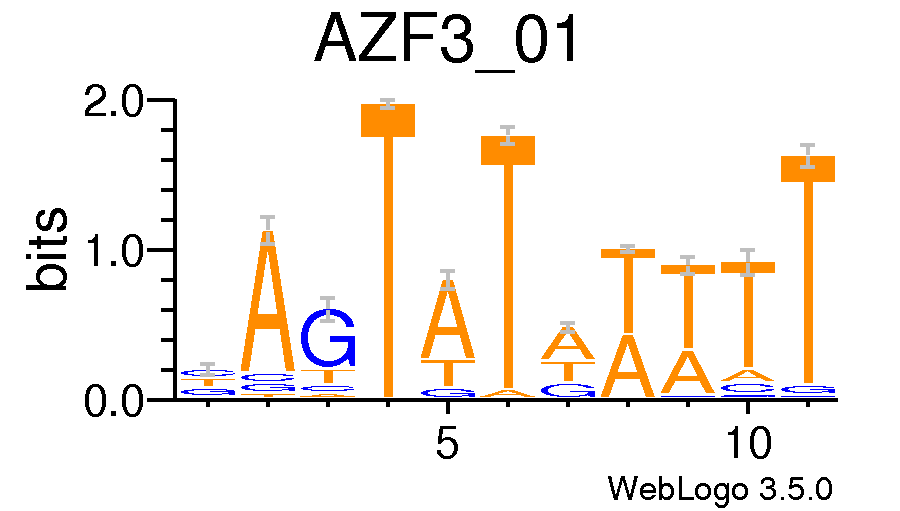 | 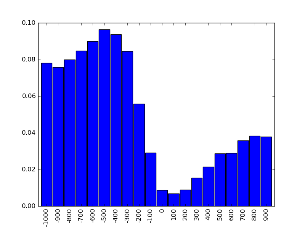 | 4.013348 |
| BZR1_02 | 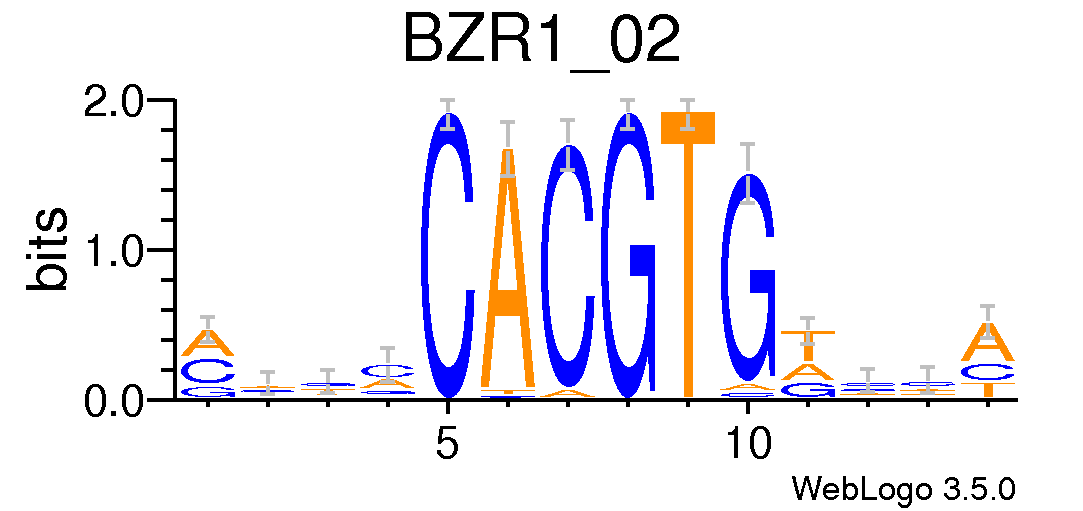 | 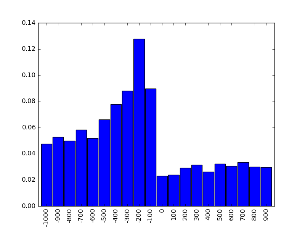 | 4.133933 |
| CBF2_03 | 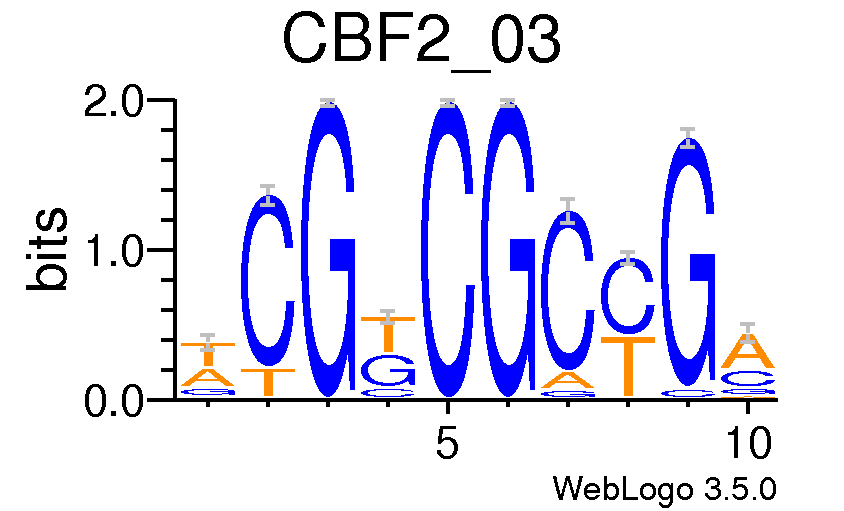 | 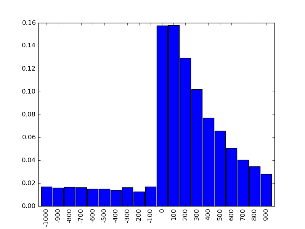 | 3.755615 |
| CG1_Q6 | 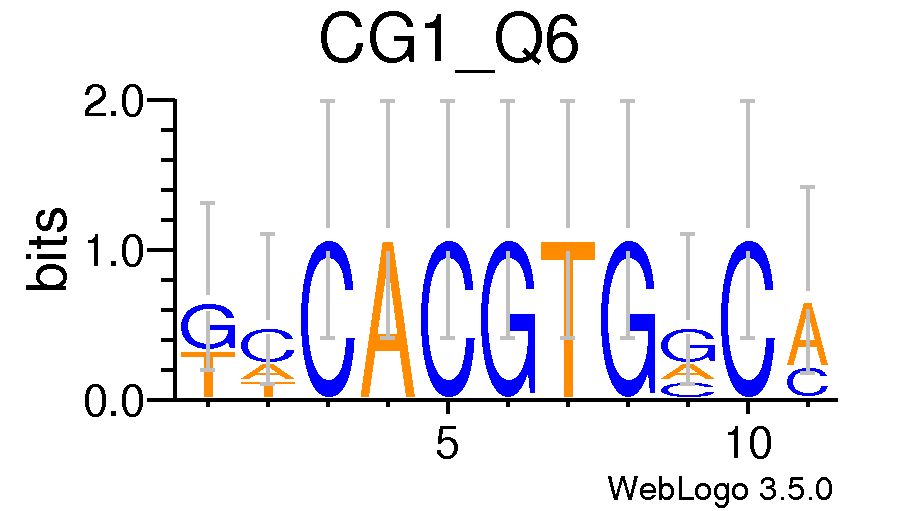 | 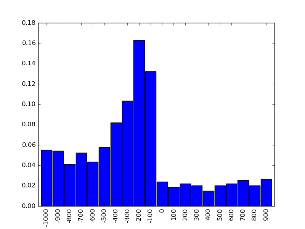 | 3.938547 |
| CPRF1_01 | 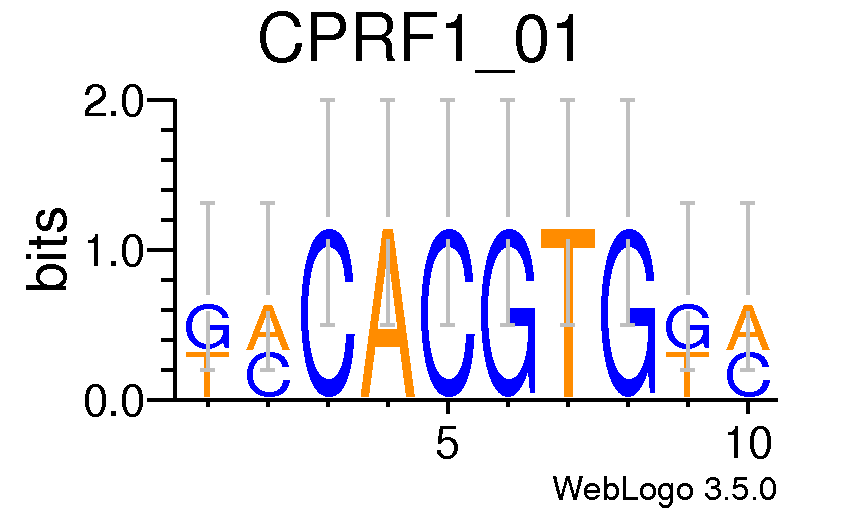 | 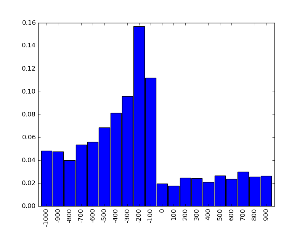 | 4.007745 |
| DAG2_01 | 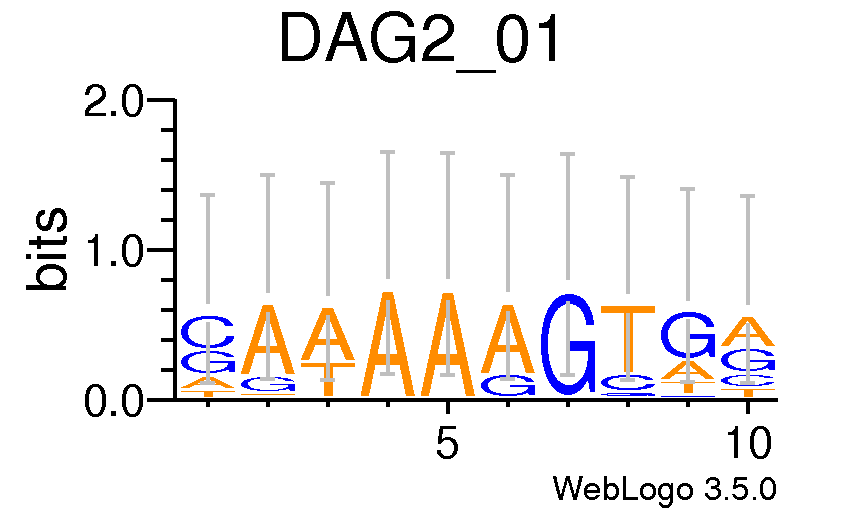 | 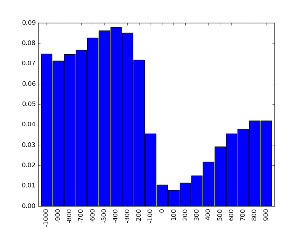 | 4.063958 |
| DREB1B_01 | 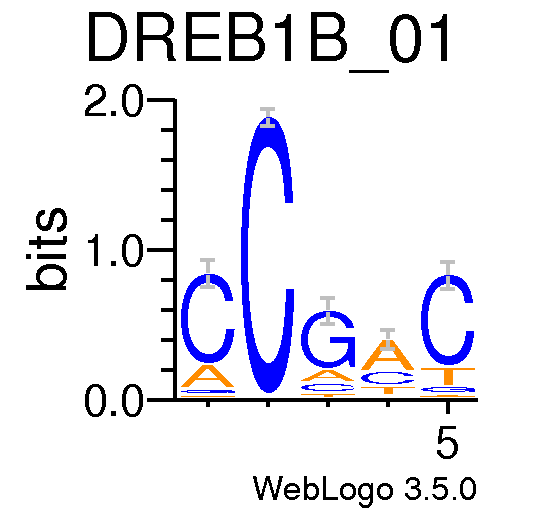 | 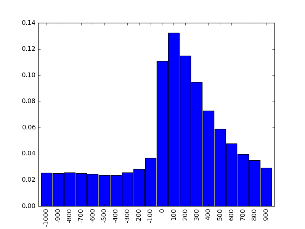 | 4.027179 |
| DREB2C_02 | 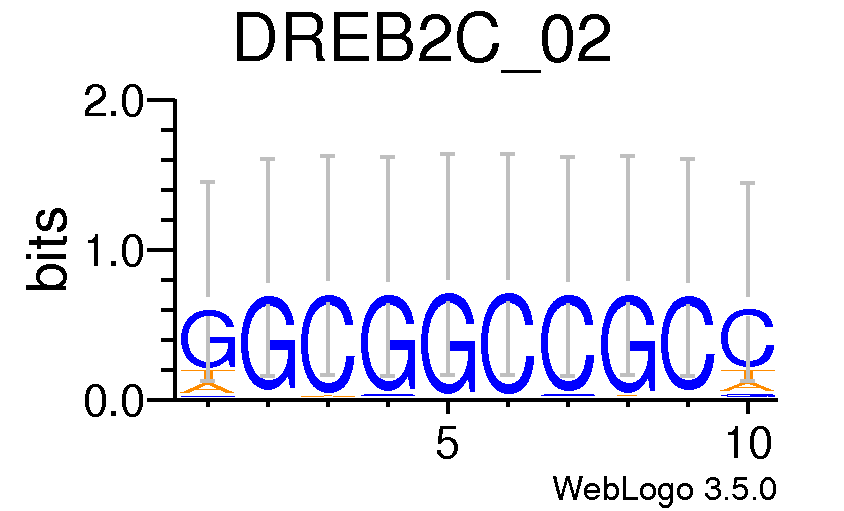 | 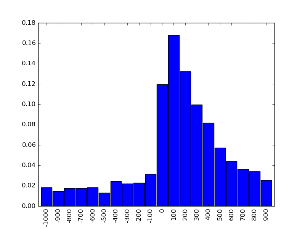 | 3.845147 |
| E2L_Q2 | 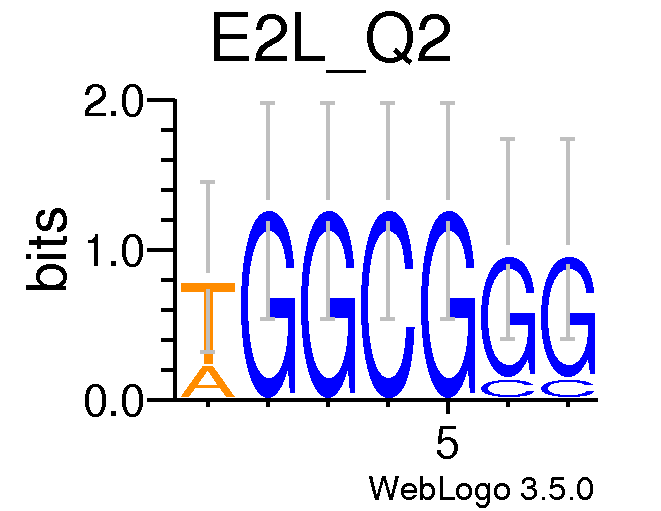 | 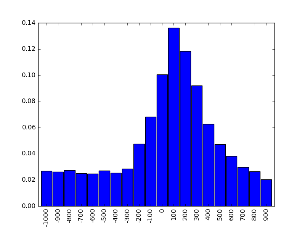 | 4.032228 |
| ERF039_01 | 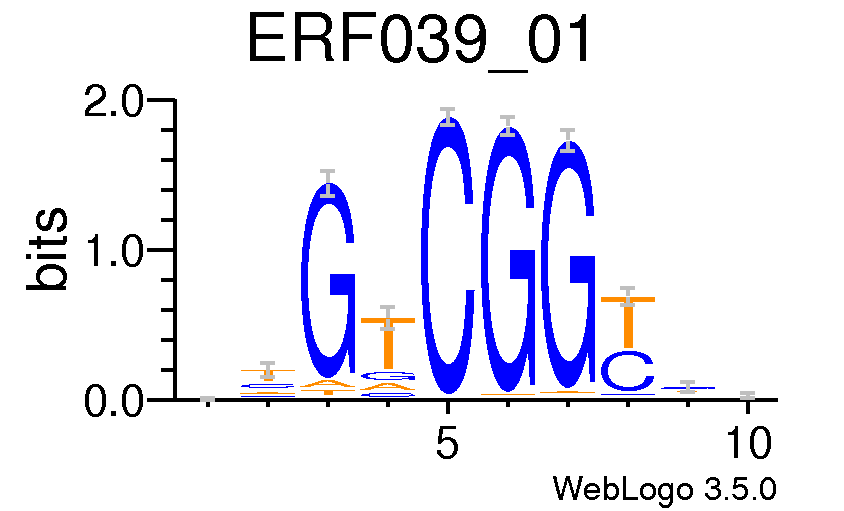 | 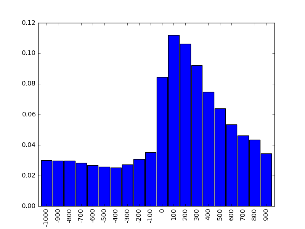 | 4.118440 |
| ERF1_Q2 | 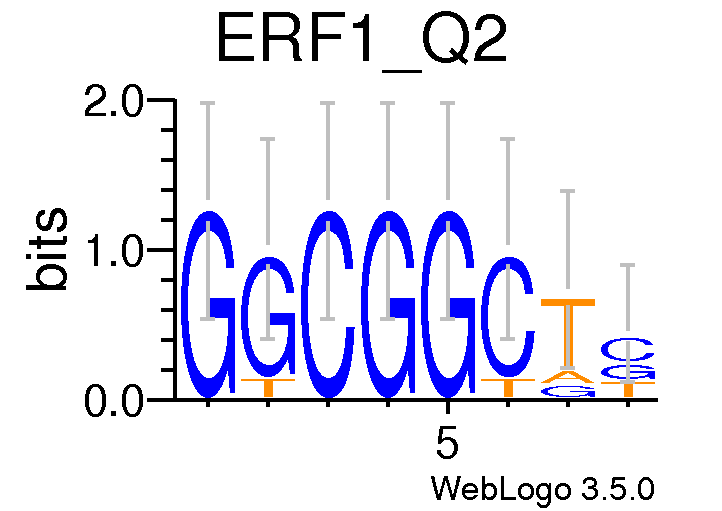 | 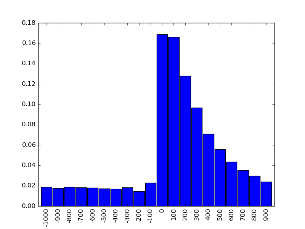 | 3.765455 |
| ERF105_02 | 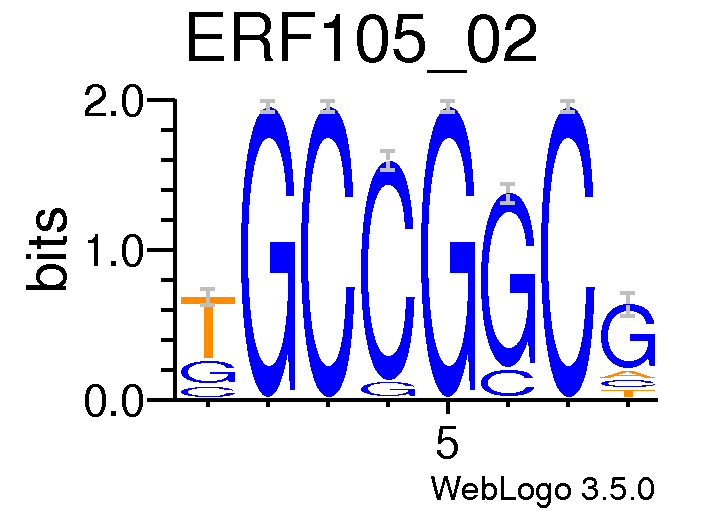 | 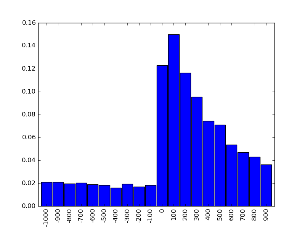 | 3.904518 |
| ERF112_01 | 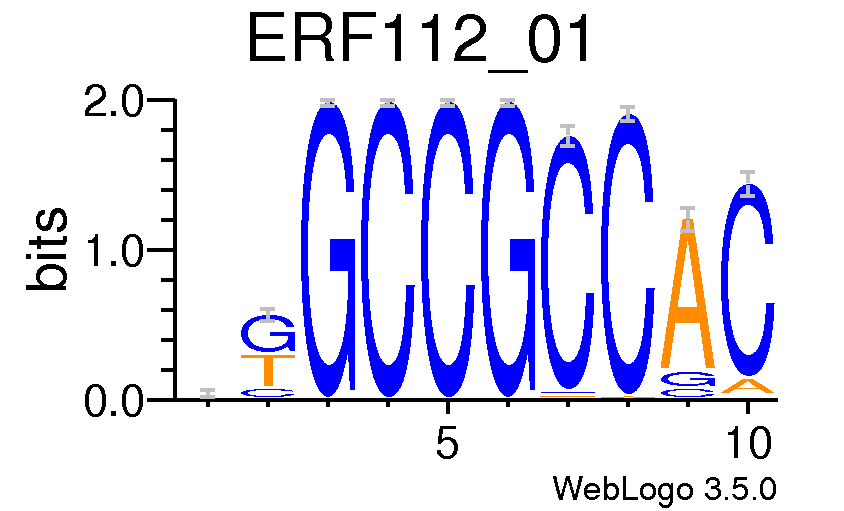 | 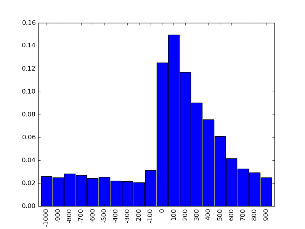 | 3.959973 |
| ERF112_02 | 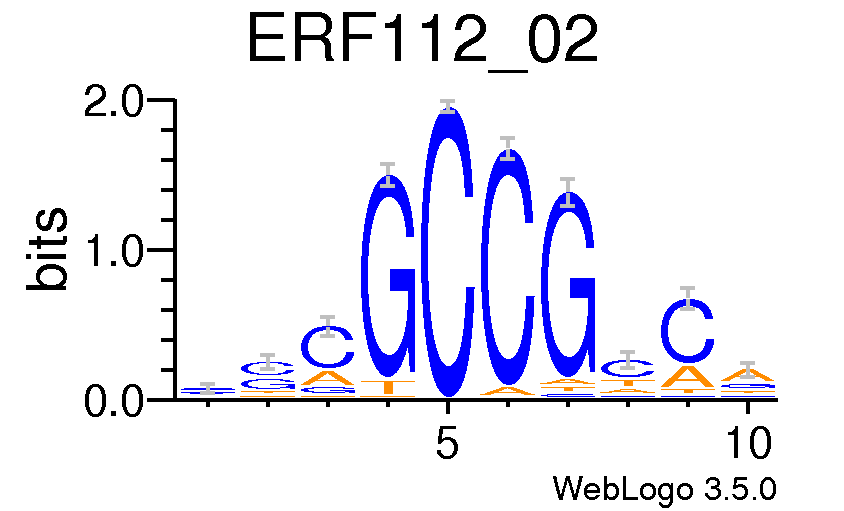 | 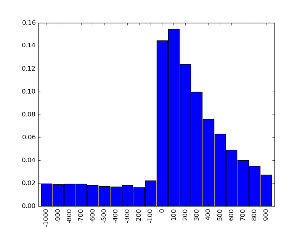 | 3.840558 |
| ERF4_04 | 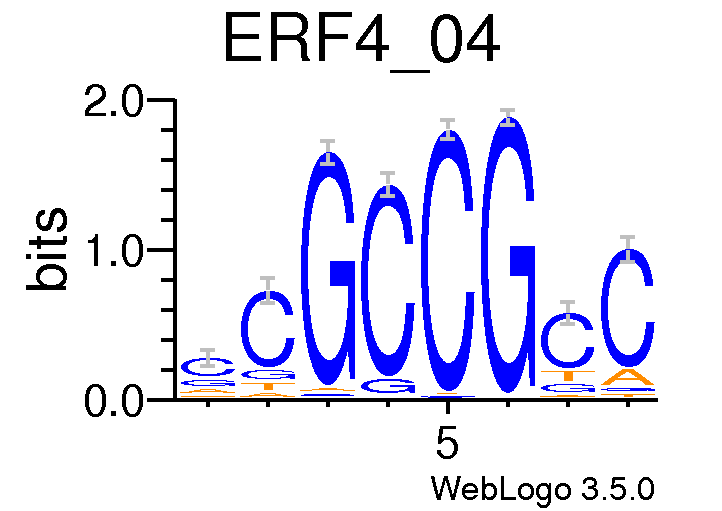 | 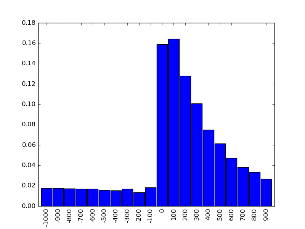 | 3.762750 |
| ERF6_02 | 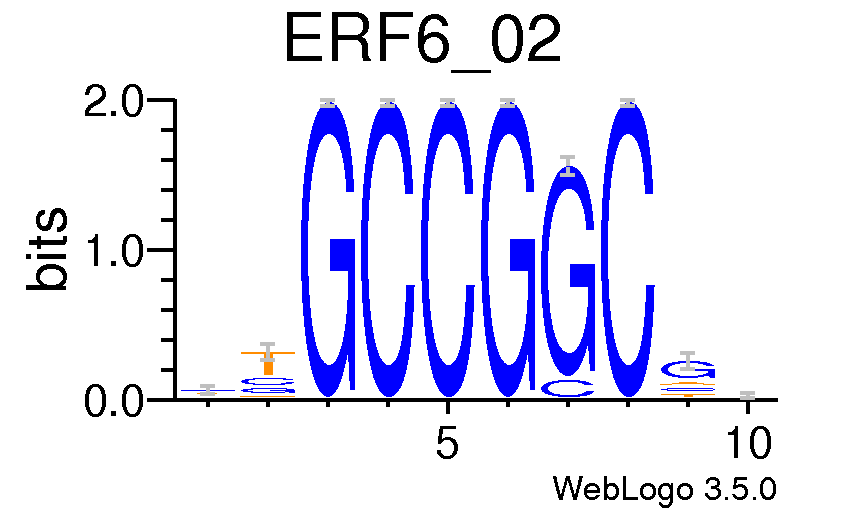 | 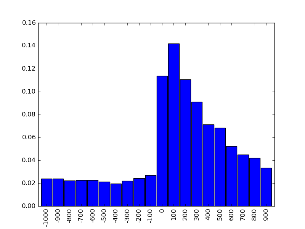 | 3.990421 |
| ERF8_01 | 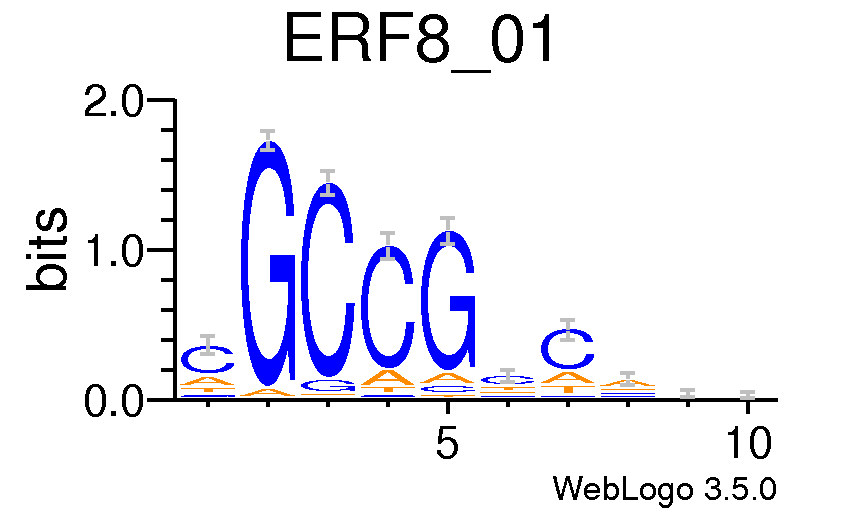 | 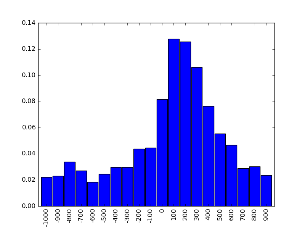 | 3.927487 |
| GBF1_Q2 | 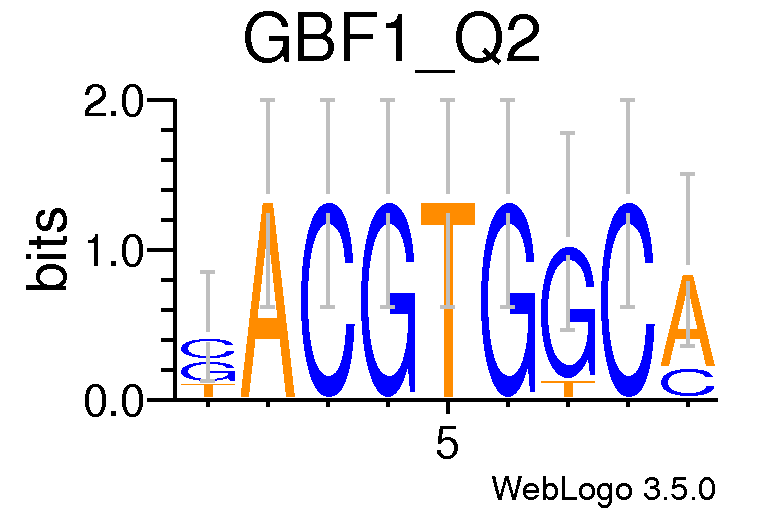 | 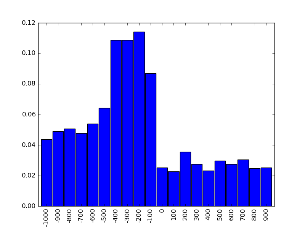 | 4.091342 |
| GT1_Q6_01 | 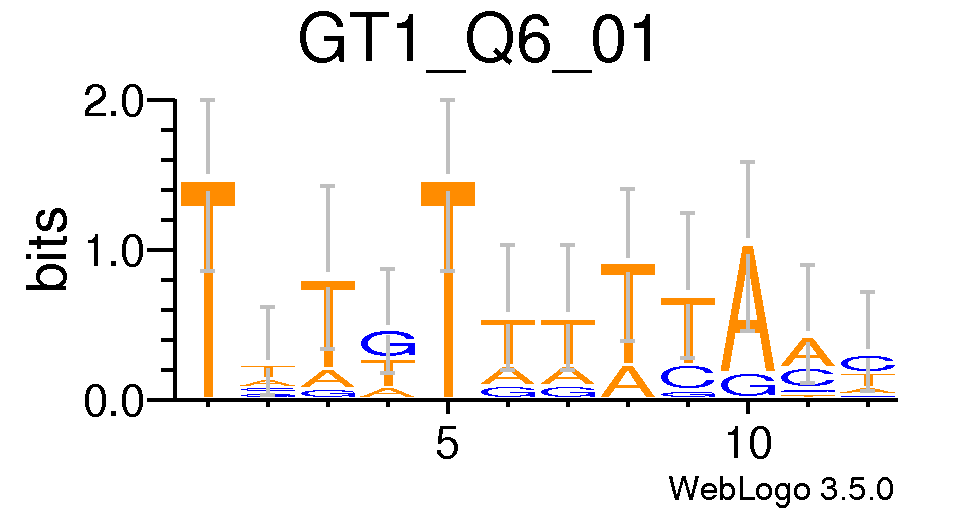 | 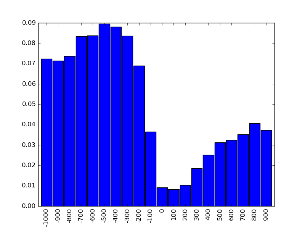 | 4.061822 |
| HAHB4_01 | 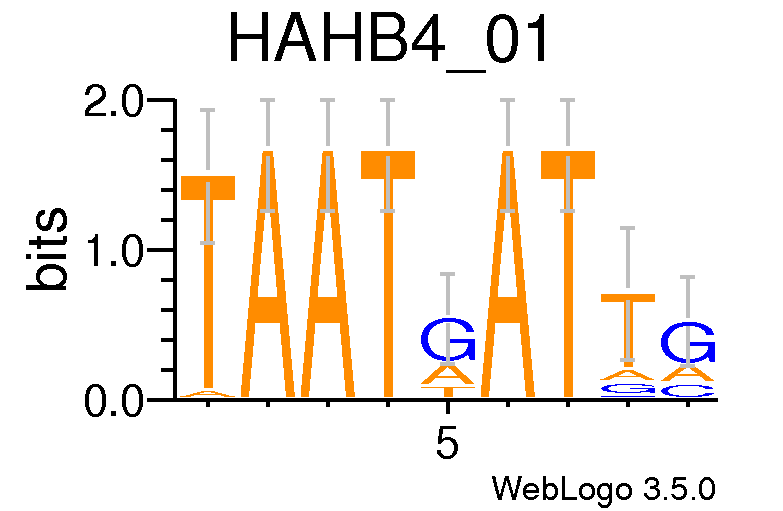 | 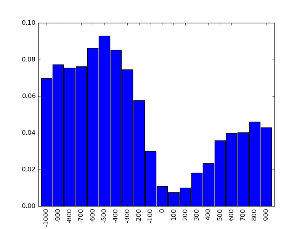 | 4.076989 |
| HBPA1_Q6_01 | 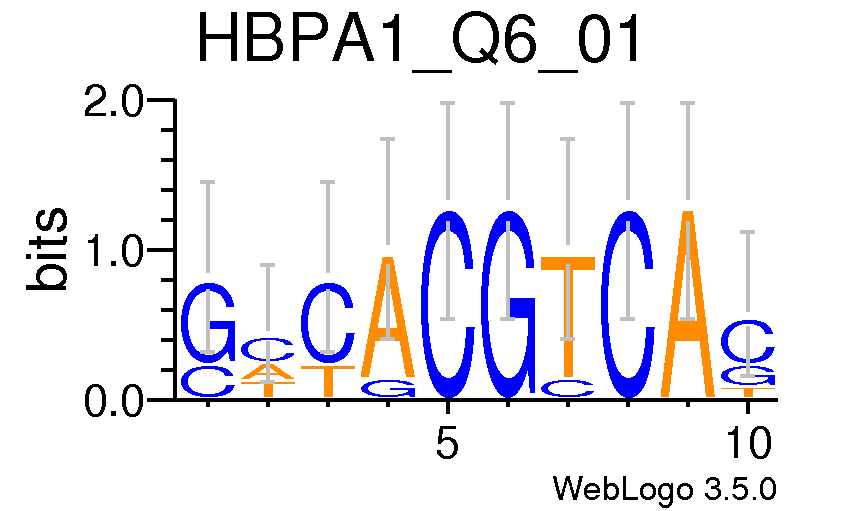 | 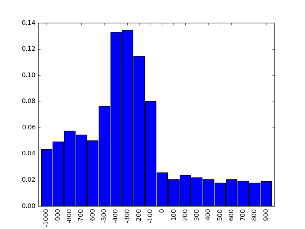 | 3.956166 |
| HSFA1E_01 | 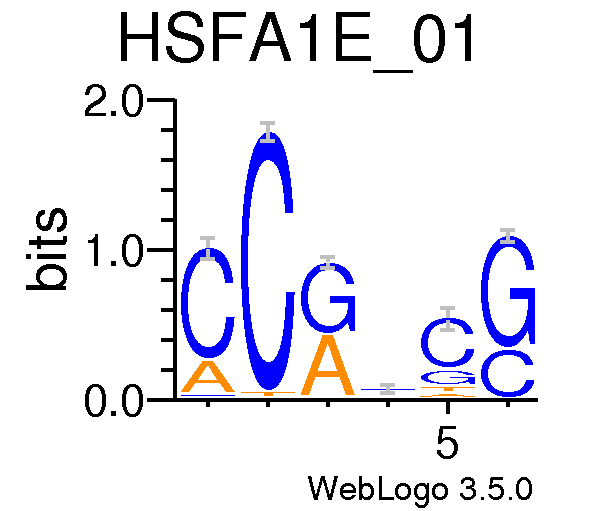 | 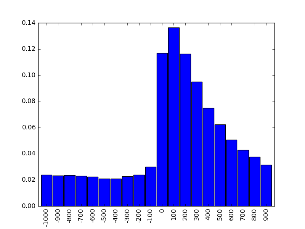 | 3.986291 |
| LIM1_01 | 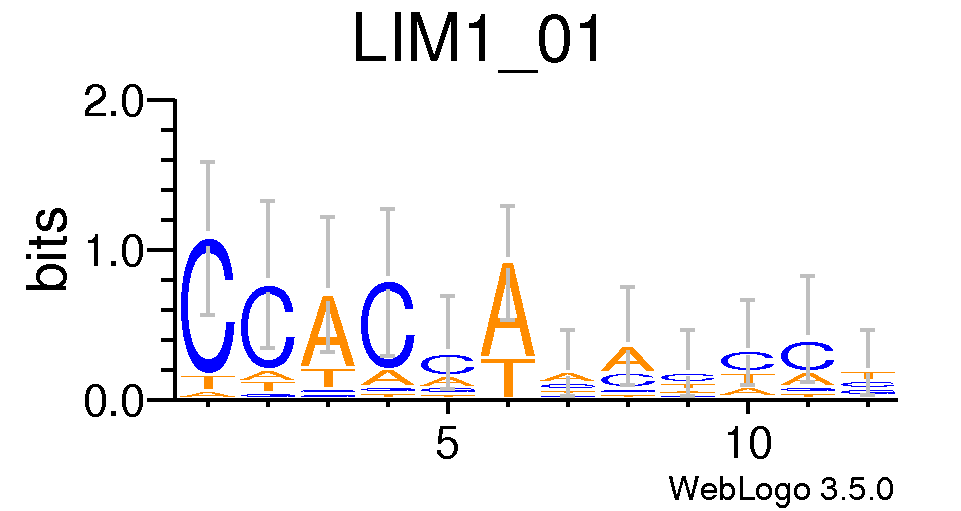 | 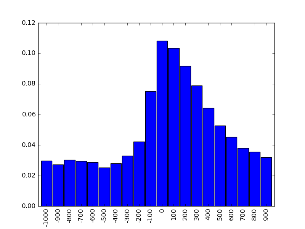 | 4.139194 |
| NAC043_01 | 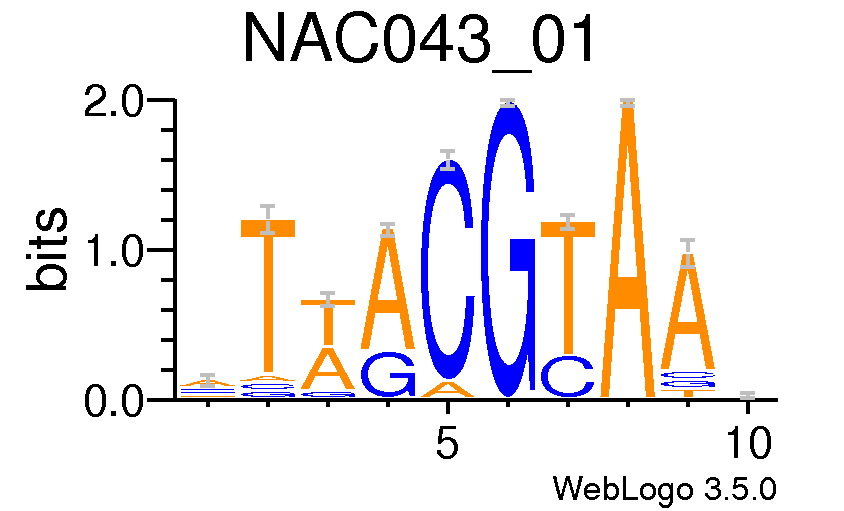 | 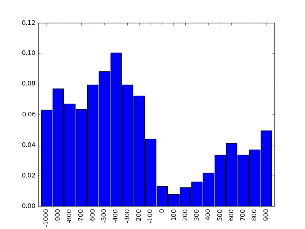 | 4.082551 |
| PCF5_01 | 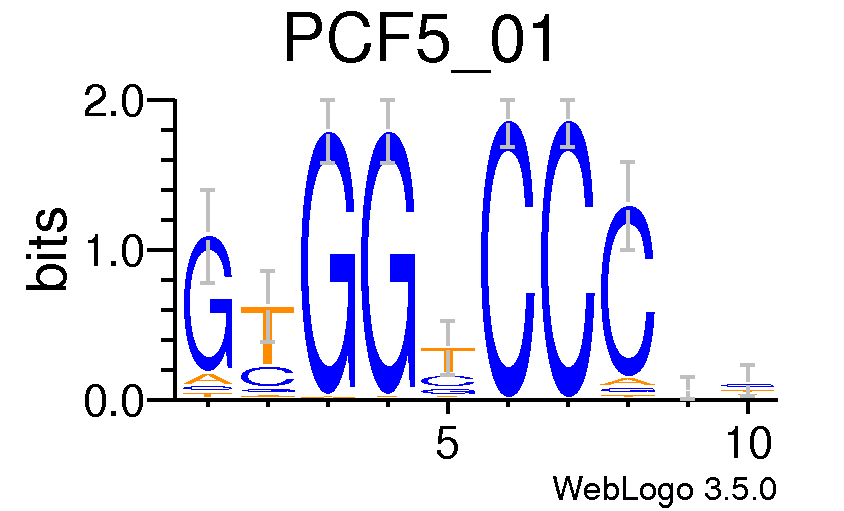 | 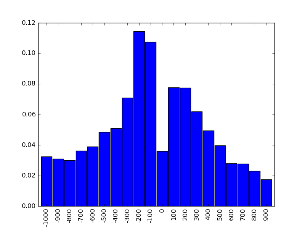 | 4.137985 |
| PEND_02 | 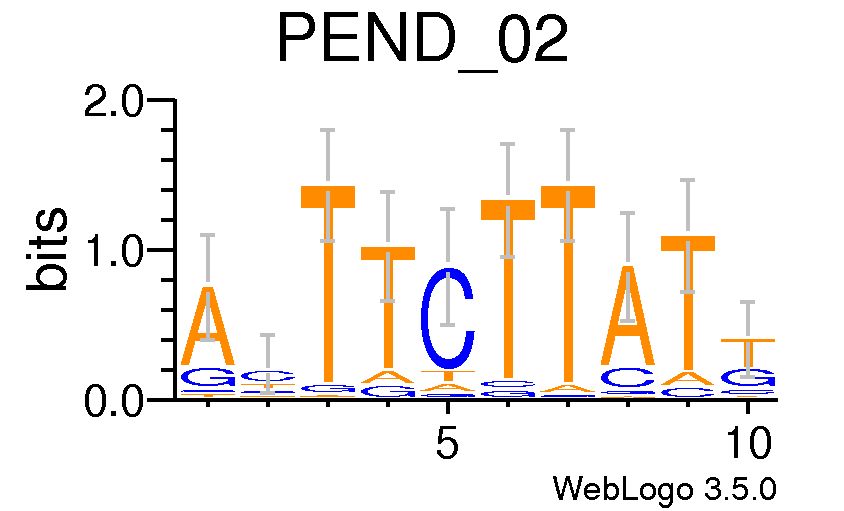 | 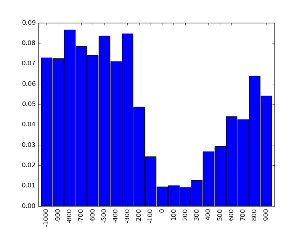 | 4.074873 |
| PIF3_02 | 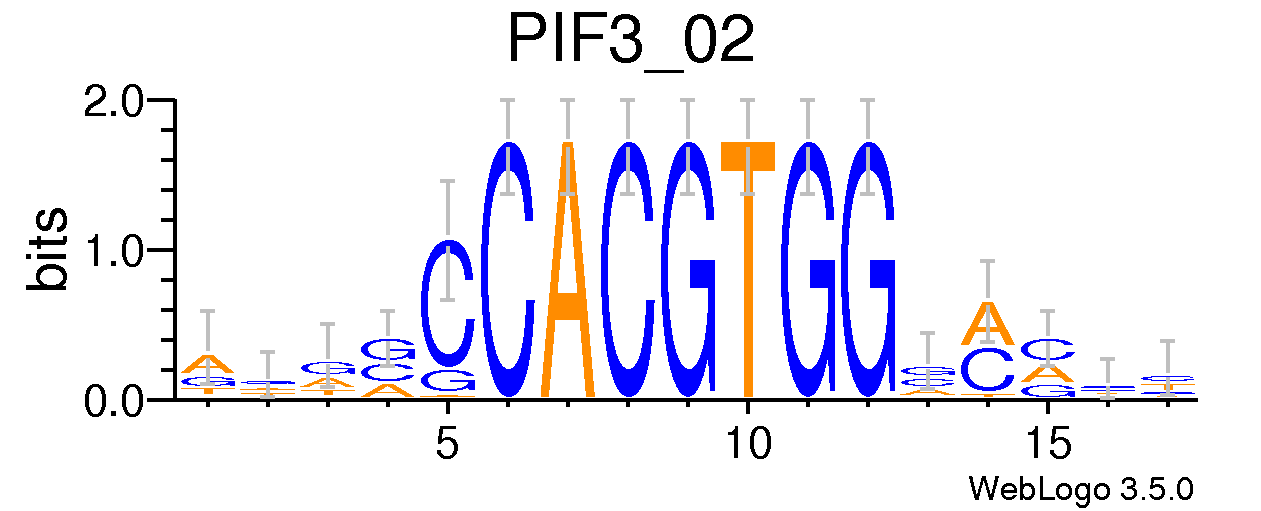 | 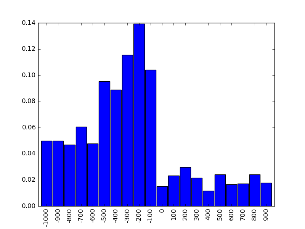 | 3.948859 |
| ROM_Q2 | 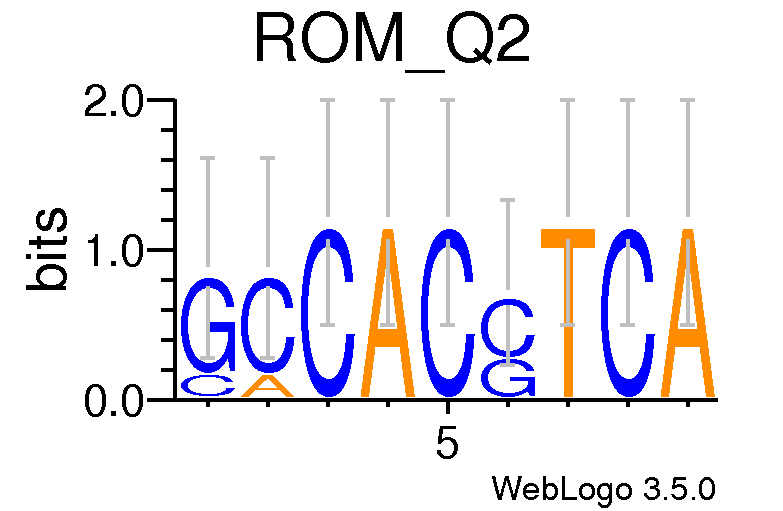 | 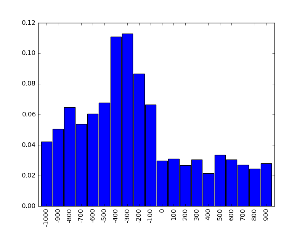 | 4.127409 |
| SBF1_01 | 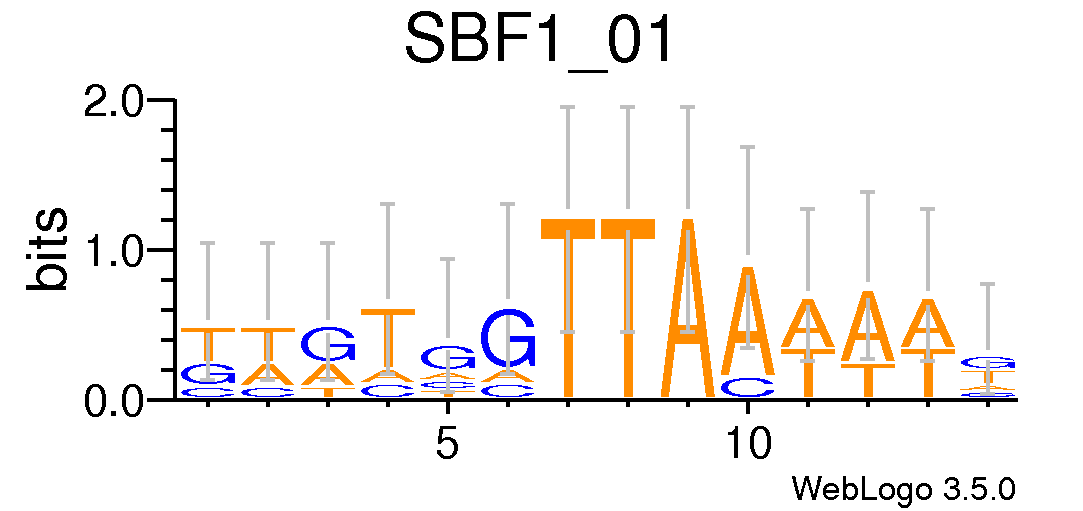 | 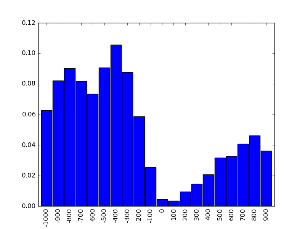 | 3.993188 |
| SPF1_Q2 | 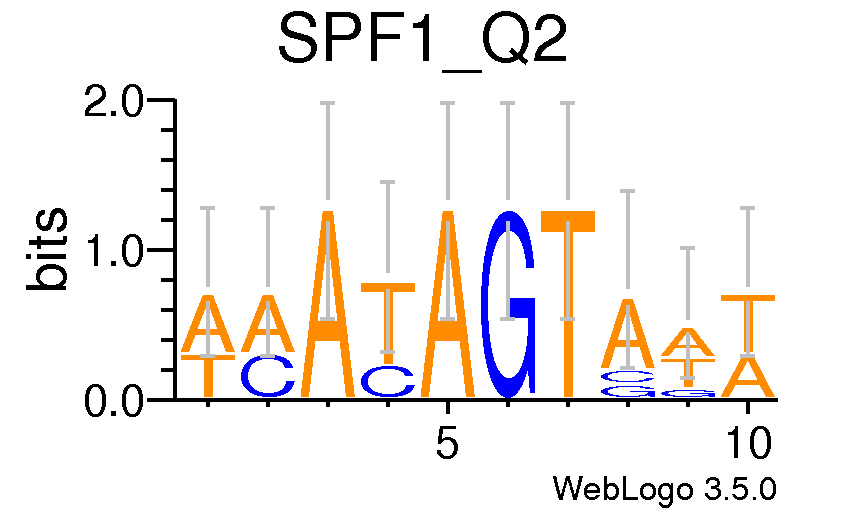 | 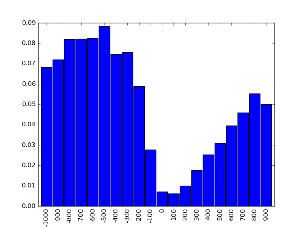 | 4.071421 |
| TCP11_01 | 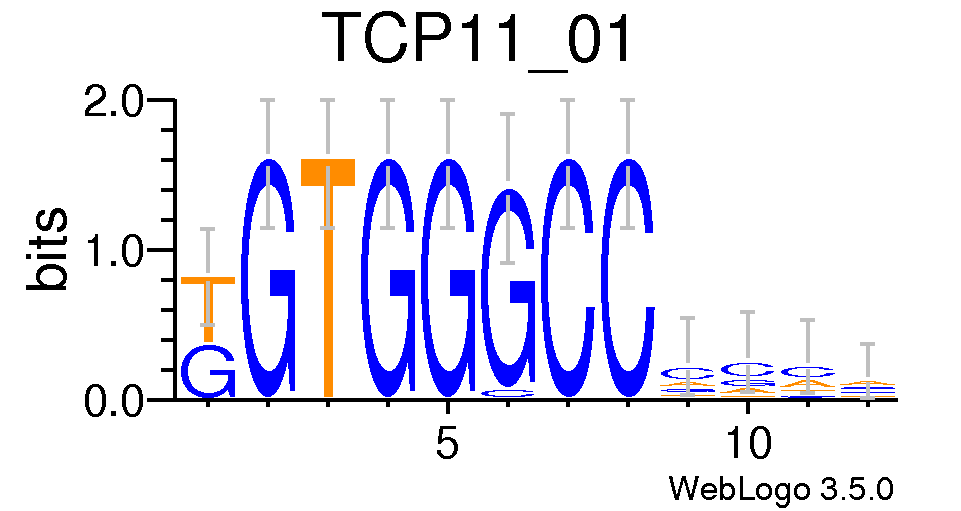 | 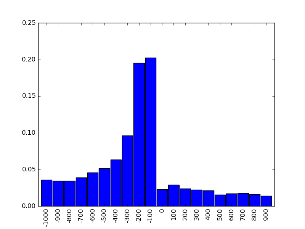 | 3.744426 |
| TCP15_02 | 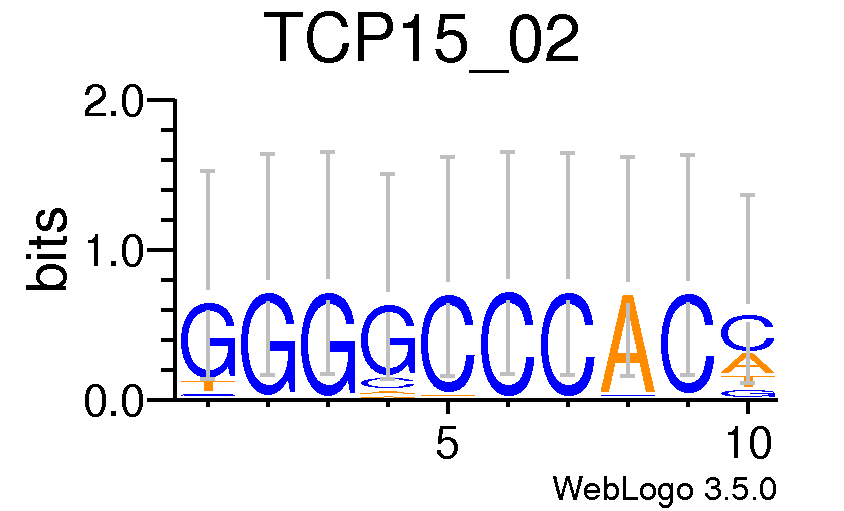 | 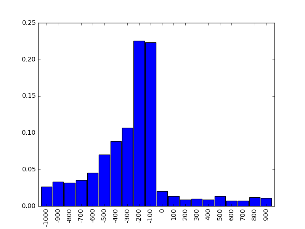 | 3.448238 |
| TCP20_02 | 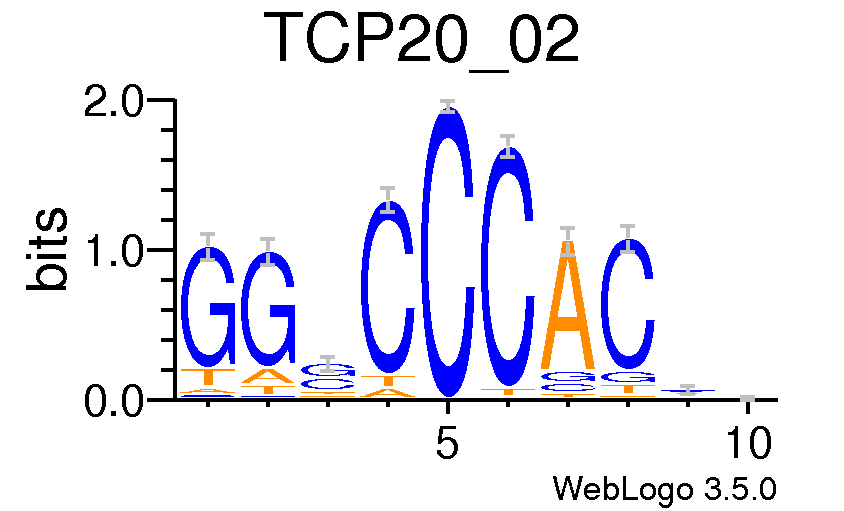 | 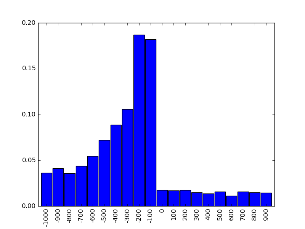 | 3.708680 |
| WRKY48_01 | 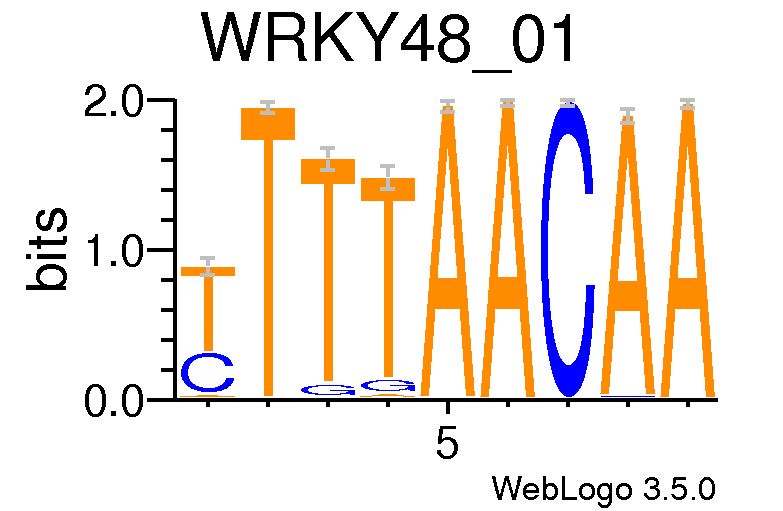 | 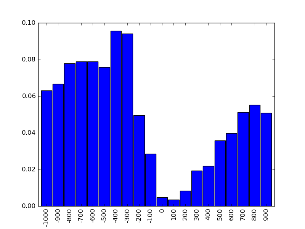 | 4.045325 |
